# Cucurbitacin B Inhibits Cell Proliferation by Regulating X-Inactive Specific Transcript Expression in Tongue Cancer

**DOI:** 10.3389/fonc.2021.651648

**Published:** 2021-07-06

**Authors:** Boqiang Tao, Dongxu Wang, Shuo Yang, Yingkun Liu, Han Wu, Zhanjun Li, Lu Chang, Zhijing Yang, Weiwei Liu

**Affiliations:** ^1^ Department of Oral and Maxillofacial Surgery, Hospital of Stomatology, Jilin University, Changchun, China; ^2^ Laboratory Animal Center, College of Animal Science, Jilin University, Changchun, China; ^3^ Jilin Provincial Key Laboratory of Oral Biomedical Engineering, Changchun, China

**Keywords:** *XIST*, miRNA, cucurbitacin B, cell proliferation, tongue cancer

## Abstract

Cucurbitacin B (CuB), a natural product, has anti-tumor effects on various cancers. In order to investigate the expression of long non-coding RNAs (lncRNA), we carried out RNA sequencing (RNA-seq) and quantitative PCR (qPCR). The data indicated that CAL27 and SCC9 tongue squamous cell carcinoma (TSCC) cells had reduced expression of X-inactive specific transcript (XIST) after CuB treatment. Moreover, our results showed increased expression of *XIST* in human tongue cancer. In this study, CuB treatment inhibited proliferation, migration and invasion of SCC9 cells, and induced cellular apoptosis. Interestingly, knockdown of XIST led to inhibition of cell proliferation and induced apoptosis *in vitro*. In addition, reduced expression of *XIST* suppressed cell migration and invasion. MicroRNA 29b (miR-29b) was identified as a direct target of *XIST*. Previous reports indicated that miR-29b regulates p53 protein. Our results suggest that increased expression of miR-29b induces cell apoptosis through p53 protein. The clustered regularly interspaced short palindromic repeats (CRISPR)/CRISPR-associated protein 9 (CRISPR/Cas9) system validated the role of *XIST* knockout in tumor development *in vivo*. Together, these results suggest that CuB exerts significant anti-cancer activity by regulating expression of *XIST via* miR-29b.

## Introduction

Although significant advances have been made in the diagnosis and treatment of TSCC, a type of oral cancer, the 5-year overall survival remains low. The natural products, including traditional Chinese medicine, have been widely used to treat human cancer (1). Recently, CuB, which is derived primarily from *Trichosanthes cucumerina L.* fruits, has been shown to be effective across various cancers. There is evidence that the anti-cancer properties of CuB are through regulated cell death by altering cell cycle progression in osteosarcoma cells (2, 3). Moreover, CuB has the ability to induce cell apoptosis *via* the Janus kinase 2/signal transducer and activator of transcription 3 signaling pathway across human gastric carcinoma cells (4). In addition, CuB inhibits cell proliferation by regulating focal adhesion kinase/p53 pathways in human cholangiocarcinoma cells (5). Previous reports indicated that CuB suppresses cell proliferation through lncRNAs and microRNAs (miRNAs) in pancreatic cancer cells (6). Results from these studies suggest that CuB plays an important role in cancer development.

A previous report suggested that numerous lncRNAs are aberrantly expressed in TSCC (7). *XIST*, a lncRNA, regulates X chromosome inactivation (XCI) (8), and plays a crucial role in the development of numerous cancers. The expression pattern of lncRNA *XIST* is associated with cellular apoptosis, proliferation, migration, and invasion in human colorectal cancer (CRC), as well as bladder cancer (9, 10). Compared to lncRNAs, miRNAs are small non-coding RNA molecules that are approximately 20 - 22 nucleotides in length. As a tumor suppressor, miR-29 inhibits cell growth and induces cell apoptosis (11). Moreover, miR-29 has a function in epithelial-mesenchymal transition (EMT) (12). There is evidence to indicate that the miR-29 family activates the p53 pathway in order to induce cell apoptosis in MCF-7 and HeLa cells (13). Indeed, lncRNAs work with miRNAs to participate in cancer development. However, the roles of *XIST* and miR-29b in TSCC have not yet been investigated.

Herein, we investigated *XIST* expression by RNA-seq in SCC9 cells after CuB treatment. Moreover, we examined the role of miR-29b as a potential target of *XIST*. In order to analyze the role of *XIST* and miR-29b in cell apoptosis, proliferation, migration and invasion, *XIST* and miR-29b were both overexpressed and knocked down. To validate the role of miR-29b in cell apoptosis, we investigated the expression of p53 protein. In addition, we evaluated the effects of CuB on tumor development in nude mice. Finally, we utilized the CRISPR/Cas9 system to knock out (KO) the *XIST* gene, and to evaluate its role in tumor development *in vivo* (14). Our findings have suggested that CuB exerts its anti-cancer effects by regulating *XIST* and miR-29b expression in TSCC.

## Materials and Methods

### TSCC Samples

All TSCC samples were acquired from patients (N = 3) which underwent clinical surgery at the Hospital of Stomatology of Jilin University. The tumor samples were collected and stored in liquid nitrogen.

### Cell Culture

CAL27 and SCC9 cells were cultured in Dulbecco’s modified Eagle’s medium (DMEM; Gibco, Gaithersburg, MD, USA), high glucose or DMEM/F12. The culture media was supplemented with 10% fetal bovine serum and 100 U/mL penicillin/streptomycin (Gibco). The cells were cultured in a 5% CO_2_ incubator at 37°C.

### RNA Isolation and RNA-Seq Analysis

In order to analyze the differential expression of lncRNAs after CuB treatment in SCC9 cells, we utilized RNA-seq. The SCC9 cells were grown to 70-90% confluency, and were seeded onto a 6-well plate and treated with CuB (Sigma, St. Louis, MO, USA) for 48 h. Next, total RNA was extracted from SCC9 cells through the use of a TRIzol reagent (Invitrogen, Carlsbad, CA, USA). Ribosomal RNA was removed and purified using the Ribo Zero Magnetic Gold Kit (Sangon Biotech, Shanghai, China). RNA-seq was performed using the Sequencing and Non-Coding RNA Program (Sangon Biotech, Shanghai, China). Next, the reads were aligned to the genome. Then, the reads were compared to known genes, and the raw counts were generated and calculated using various algorithms, including HISAT2, RSeQC, BEDTools and Qualimap.

### Knockdown and Overexpression of *XIST* and miR-29b

For knockdown, the small interfering RNA (siRNA) target sequence of *XIST* (GCATGCATCTTGGACATTT) was purchased from RiboBio (Guangzhou, China), while pcDNA3.1-XIST was purchased from GenePharma (Shanghai, China) for overexpression. The miR-29b-3p-mimics and miR-29b-3p-inhibitor were purchased from RiboBio. The SCC9 cells were transiently transfected with pcDNA3.1-XIST, si-XIST, miR-29b-3p-inhibitor or miR-29b-3p-mimics for 48 h *in vitro*. A non-specific siRNA was transfected into control (Con) cells.

### Construction of pcDNA3.1-XIST and XIST-KO Cells Lines by Stable Transfection

In order to construct the pcDNA3.1-XIST overexpression cell line, we utilized SCC9 cells. The SCC9 cells were cultured without FBS once cells reached a confluence of 80% for 12 – 16 h. Next, the pcDNA3.1-XIST (2 μg) and Lipofectamine 2000 (Invitrogen, USA) were utilized for transfection. After incubation for 48 h, G418 (400 mg/mL, Invitrogen, USA) was added into SCC9 cells. The clones were grown and picked after 14 days.

The CRISPR/Cas9 plasmid (px459; Addgene, Watertown, MA, USA) was utilized for gene editing of *XIST.* The design of a single guide RNA (sgRNA) has been previously described (14). The sgRNAs and primer sequences are listed in **Table S1**. Plasmid construction with sgRNA (XIST-KO) was validated *via* sequencing (Sangon, China). Transfection was conducted using XIST-KO (5 μg) and Lipofectamine 2000 (Invitrogen, USA) for 48 h. Next, puromycin was used to select the positive clones. After 14 days, the clones (XIST-KO) were grown and picked for subsequent western blot, qPCR and sequencing analysis.

### Analysis of Gene Expression

Total RNA was isolated from CAL27 and SCC9 cells. Additionally, total RNA was extracted from human tumor and mouse tumor samples through the use of TRIzol reagents (Invitrogen, Carlsbad, CA, USA). Using the BioRT cDNA first-strand synthesis kit (Bioer Technology, Hangzhou, China), cDNA was generated as per the manufacturer’s protocols. The qPCR was carried out to detect the expression of genes utilizing the BioEasy SYBR Green I Real-Time PCR Kit (Tiangen, Beijing, China). The qPCR reaction was carried out at 94°C for 3 min, followed by 35 denaturation cycles at 94°C for 10 s, annealing at 59°C for 15 s, and extension at 72°C for 30 s. *GAPDH* was utilized as an internal reference. The 2^-ΔΔCT^ method was used to assess relative gene expression. All experiments were conducted in triplicate.

### Western Blot

The protein from TSCC cells was extracted utilizing the protein extraction buffer (Beyotime, China). In order to quantify the proteins from TSCC cells, we utilized the BCA protein assay kit (Tiangen, China). The proteins were then separated through the use of 10% SDS-PAGE gels. Next, these proteins were transferred onto the PVDF membranes. These membranes were then blocked (5% non-fat milk powder) and washed with TBST (0.1% Tween-20). The primary antibodies, including anti-p53 (Abcam, ab131442, USA), anti-E-cadherin (Proteintech, 20874-1-AP, USA) and anti-Gapdh (Bioworld, AP0066, USA), were incubated with the membrane overnight at 4°C. Subsequently, the membranes were incubated with HRP-conjugated affiniPure goat antibodies IgG (BOSTER, China) for 2 h. Finally, the proteins were identified using ECL Super Signal (Pierce; Thermo Fisher Scientific, USA).

### The Colony Formation Assay

For the colony formation assay, the target cells (1 × 10^3^) were seeded per well of a 60 mm plate. After 2 weeks, colonies were fixed with paraformaldehyde and stained with 0.1% (w/v) crystal violet. Photographs were acquired and the cell numbers were counted.

### Cell Migration Analysis

Cell migration was measured through the use of a wound healing assay. In brief, 5 × 10^5^ cells were seeded and cultured 48 h prior to transfection. A scraped line was created, and cells were cultured in a serum-free medium. The scratched area of cell migration was then visualized at 12, 24, and 48 h. Through the use of an inverted microscope and ImageJ software, the cell migration area was measured. All experiments were conducted in triplicate.

### Cell Invasion Analysis

The cell invasion was analyzed through the use of a Transwell assay (15). In brief, SCC9 cells were treated with CuB or transfected with pcDNA3.1-XIST, si-XIST, miR-29b-3p-inhibitor or miR-29b-3p-mimics. Next, they were serum-starved for 24 h. Subsequently, cells (3 × 10^4^) were added to the upper chamber of Transwell assay (Corning, New York, NY, USA), which contained 20 μL of Matrigel (BD Biosciences, Franklin Lakes, NJ, USA). This was then followed by the addition of 0.5 mL medium containing 10% FBS to the bottom chamber (Corning, New York, NY, USA). After incubation at 37°C for 24 h, cells on the bottom side were fixed in 4% paraformaldehyde and stained using a 0.1% crystal violet dye (Solarbio, Beijing, China). Using an inverted microscope and ImageJ software, the stained cells were counted. All experiments were conducted in triplicate.

### Cell Counting Kit-8 Assay

Cell viability was determined through the use of a Cell Counting Kit-8 (CCK-8) assay (Dojindo, Kumamoto, Japan). In brief, cells (4 × 10^3^) were seeded after they have undergone CuB treatment or were transfected with pcDNA3.1-XIST, si-XIST, miR-29b-3p-inhibitor, miR-29b-3p-mimics. Next, 10 μL of the CCK-8 solution was added to 96-well plates. The cells were then incubated at 37°C for 2.5 h. The absorbance (optical density) at 450 nm was utilized to measure cell viability. To analyze the CuB concentration in TSCC cells, half maximal inhibitory concentration (IC50) was performed using GraphPad Software (USA).

### Cell Apoptosis Analysis

Cell apoptosis was assessed as previously described (16). In brief, SCC9 cells were treated with CuB or transfected with pcDNA3.1-XIST, si-XIST, miR-29b-3p-inhibitor or miR-29b-3p-mimics for 48 h. The cells were then washed twice with phosphate-buffered saline. Then, the cells (1 × 10^6^ cells/mL) were harvested, and a mixture of Annexin V-FITC/PI reagent (Beyotime, China, Cat No. C1062L) was added to the cells and incubated for 30 min. Finally, cell fluorescence was analyzed through flow cytometry (BD Biosciences, USA).

### Animals and Animal Care

Overall, 36 nude mice (half male half female, 8 weeks old) were utilized for this study. All mice were acquired from the Laboratory Animal Center of Jilin University. The mice were then grouped and housed in laboratory cages under specific pathogen-free conditions at a temperature of 24°C, with a relative humidity of 50 - 60%, and under a 12-h-light/12-h-dark schedule. Animals were then provided *ad libitum* access to standard rodent food and tap water. All mice were healthy and not infected during the experimental period. All surgical procedures were carried out under aseptic conditions. The SCC9 cell lines (8 × 10^6^) were subcutaneously injected into the left flank of each mouse, and tumors were observed after seven or eight days. The mice were then used for CuB treatment when tumor volumes had reached 100 - 150 mm^3^. The nude mice were treated with CuB (0.5 mg/kg, N = 6) each day for 14 days, while the Con group (N = 6) were treated with PBS. The pcDNA3.1-XIST and XIST-KO cells lines were acquired through a stable transfection. Then, the pcDNA3.1-Con, pcDNA3.1-XIST and XIST-KO cells lines (8 × 10^6^) were inoculated into nude mice. Half of pcDNA3.1-XIST group (N = 6) was administered 0.5 mg/kg of CuB. The Con (N = 6), XIST-KO (N = 6) group and half of pcDNA3.1-XIST group (N = 6) received an equivalent volume of PBS. The tumor length (L) and width (W) were recorded and tumor volumes were calculated as L×W^2^/2.

### Statistical Analysis

The unpaired Student’s t-test was utilized to assess differences between the two groups (Con and experimental group) in CCK8 assay, cell apoptosis, migration, invasion, qPCR and tumor volumes. The measurement data were tested for normal distribution using the Kolmogorov Smirnov (K-S) test to compare cumulative distribution. Data conforming with the normal distribution were summarized with mean values and standard deviations (SD). Statistical analysis was conducted using GraphPad Prism 5.0 (GraphPad Software, Inc). P < 0.05 was considered statistically significant.

## Results

### Effects of CuB in Cell Apoptosis, Growth, Migration, and Invasion

**Figure 1:**
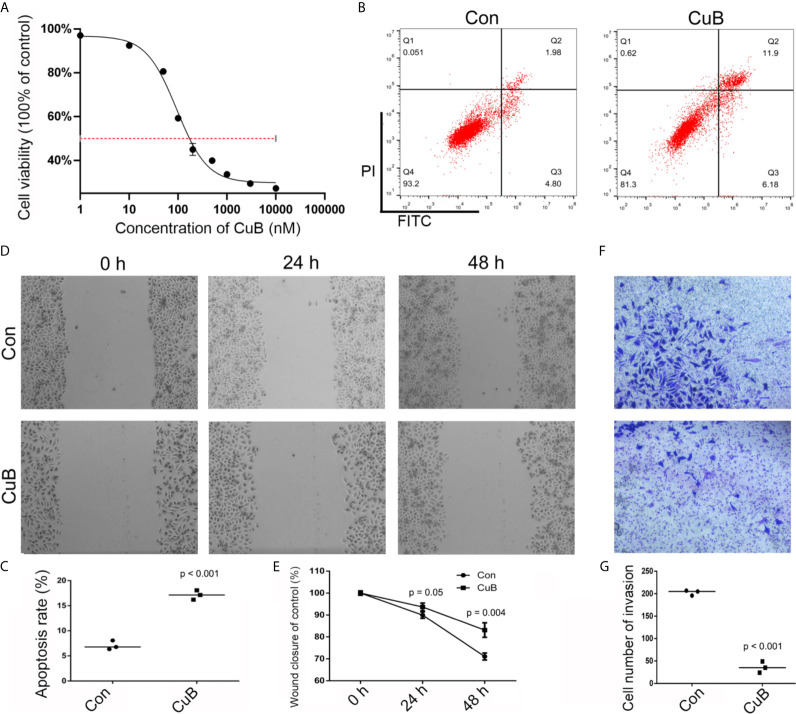
Effects on cell growth, apoptosis, migration, and invasion after CuB treatment. **(A)** IC50 was assessed in SCC9 cells after CuB treatment to measure the proliferation of SCC9 using CCK8. **(B)** Cell apoptosis was analyzed in the Con and CuB groups (50 nM) in SCC9 cells to determine the effect of CuB on cell apoptosis. **(C)** Statistical analysis of the percentage of cell apoptosis among the two groups. **(D)** Analysis of cell migration in the Con and CuB groups in SCC9 cells to determine the effect of CuB on cell migration. **(E)** Statistical analysis of the percentage of cell migration between the Con and CuB groups. **(F)** Analysis of cell invasion in the SCC9 cells to determine the effect of CuB on cell invasion. **(G)** Statistical analysis of the percentage of cell invasion migration between the Con and CuB groups. Statistical significances between groups were evaluated using t-test for independent groups.

In order to analyze the viability of SCC9 cells, the CCK-8 assay was conducted after CuB treatment. IC50 data indicated that 200 and 500 nM CuB had toxic effects on SCC9 cells (**Figure 1A**). Thus, 50 nM CuB was utilized for qPCR analysis. Moreover, compared to Con cells, CuB was able to induce apoptosis (**Figures 1B, C**), and inhibit cell migration (**Figures 1D, E**) and invasion (**Figures 1F, G**) in SCC9 cells. Overall, these results suggest that CuB exerts its anti-cancer effects by inhibiting the migratory and invasive potential of TSCC cells.

### Expression Profile of lncRNAs

**Figure 2:**
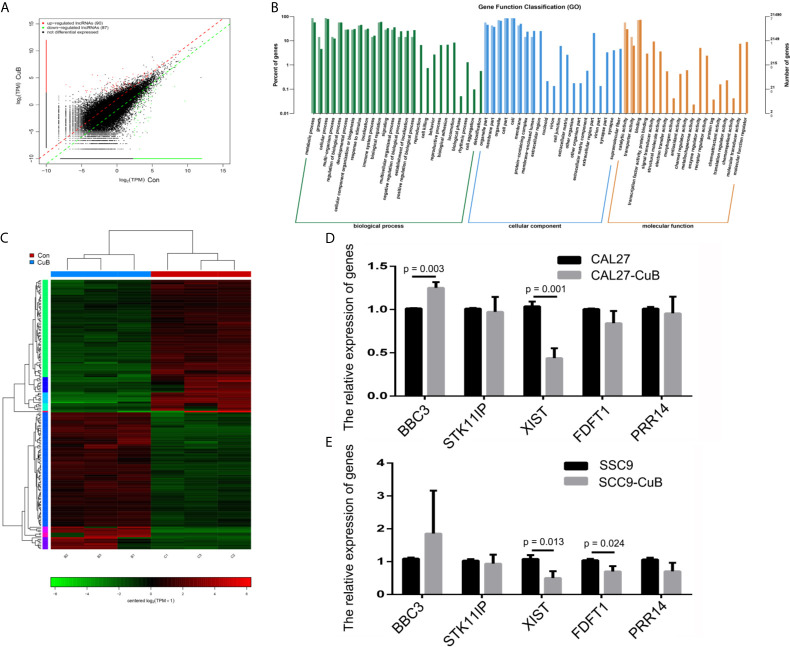
LncRNA expression profile by RNA-seq. **(A)** Identification of differentially expressed lncRNAs in SCC9 cells. **(B)** KEGG pathways associated with the differentially expressed lncRNAs. **(C)** Heatmap depicts the differentially expressed lncRNAs. **(D)** Relative expression of *BBC3, STK11IP, XIST, FDFT1* and *PRR14* were analyzed *via* qPCR post-CuB treatment of CAL27 cells. **(E)** Relative expression of *BBC3, STK11IP, XIST, FDFT1* and *PRR14* were analyzed in SCC9 cells. Data are expressed as mean ± SD (n = 3). Statistical significances between groups were evaluated using t-test for independent groups.

Next, we performed RNA-seq to determine the expression profile of lncRNAs in SCC9 cells. The results demonstrated that there were 90 upregulated and 87 downregulated lncRNAs in the CuB-treated group compared to the Con group (**Figure 2A**). Moreover, gene function classification indicated that the differentially expressed lncRNAs were more likely to be expressed during cell growth (**Figures 2B, C**). Specifically, we analyzed the role of five lncRNAs (*BBC3, STK11IP, XIST, FDFT1*, and *PRR14*), which are known to be related to cell growth and apoptosis in cancer development (**Figure S1**). The qPCR results demonstrated that *XIST* expression was reduced in both CAL27 and SCC9 cells after CuB treatment (**Figures 2D, E**). Hence, *XIST* was further analyzed in this study.

### CuB Reduced Expression of *XIST* in TSCC Cells

**Figure 3:**
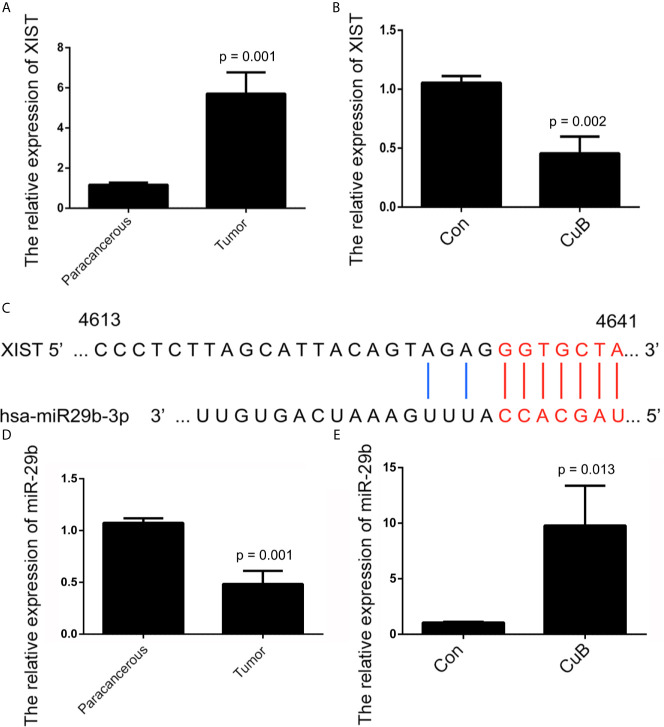
Analysis of *XIST* and miR-29b expression after CuB treatment. **(A)** Relative expression of *XIST in* TSCC patients (N = 3) using qPCR. **(B)** The relative expression of *XIST*, analyzed by qPCR, after CuB treatment of SCC9 cells. **(C)** Schematic representations of the sequences of *XIST* and miR-29b. **(D)** Relative expression of miR-29b among TSCC patients (N = 3). **(E)** Relative expression of miR-29b, analyzed by qPCR, after CuB treatment of SCC9 cells. Data are expressed as mean ± SD (n = 3). Statistical significances between groups were evaluated using t-test for independent groups.

The Cancer Genome Atlas (TCGA) database showed that higher *XIST* expression is associated with a poorer prognosis for patients with head and neck squamous cell carcinoma (HNSCC) (**Figure S2**). The qPCR results revealed that *XIST* was significantly higher expressed in TSCC tissue compared to the paracancerous tissue (**Figure 3A**). Moreover, expression of *XIST* was reduced after CuB treatment (**Figure 3B**). Next, miR-29, which is located on exon 1 of *XIST*, was investigated as a potential target of *XIST* (**Figures 3C**, **S3**). The inhibited expression of miR-29 was observed in TSCC patients (**Figure 3D**). Additionally, CuB increased miR-29b expression in SCC9 cells (**Figure 3E**). These results indicate that CuB is able to regulate expression of *XIST* and miR-29b in TSCC cells.

### Effects of *XIST* and miR-29b Expression on Cell Apoptosis, Growth, Migration, and Invasion

**Figure 4:**
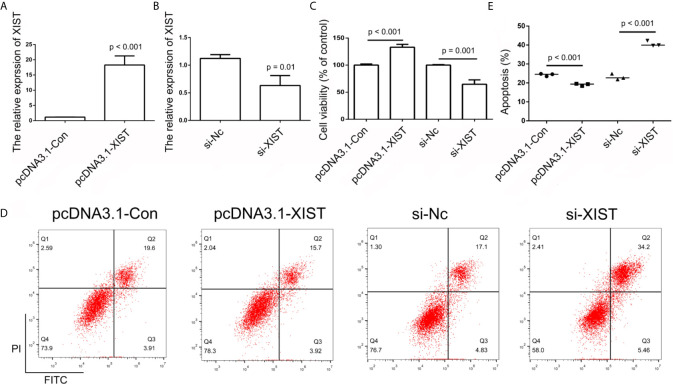
Analysis of *XIST* expression. **(A, B)** Relative expression of *XIST* in pcDNA3.1-Con, pcDNA3.1-XIST, si-Nc, and si-XIST groups, respectively, using qPCR in SCC9 cells. Data are represented as mean ± SD (n = 3). **(C)** Cell growth was analyzed using the CCK-8 assay in SCC9 cells to determine the effect of XIST on cell proliferation. **(D)** Cell apoptosis was analyzed after transfection with pcDNA3.1-XIST and si-XIST in SCC9 cells to determine the effect of XIST on cell apoptosis. **(E)** Statistical analysis of the percentage of cells undergoing apoptosis in the four groups. Statistical significances between groups were evaluated using t-test for independent groups.

**Figure 5:**
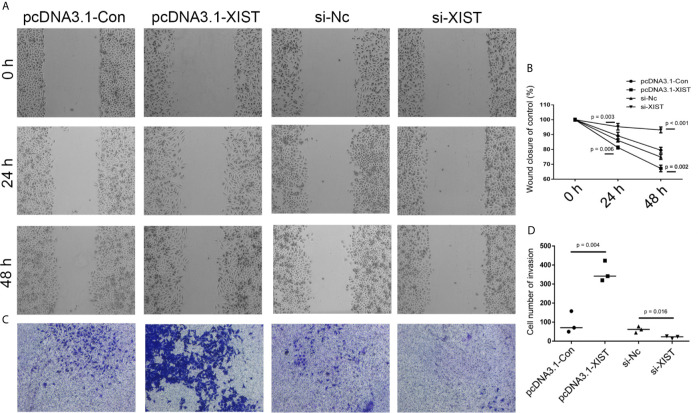
Analysis of cell migration and invasion after *XIST* knockdown and overexpression. **(A)** Cell migration was assessed in the pcDNA3.1-Con, pcDNA3.1-XIST, si-Nc, and si-XIST groups in SCC9 cells to determine the effect of XIST on cell migration. **(B)** Statistical analysis of the percentage of cell migration among the four groups in 0 h, 24 h and 48 h. **(C)** Cell invasion was assessed in the pcDNA3.1-Con, pcDNA3.1-XIST, si-Nc, and si-XIST groups in SCC9 cells to determine the effect of XIST on cell invasion. **(D)** Statistical analysis of the percentage of cell invasion among the four groups. Statistical significances between groups were evaluated using t-test for independent groups.

**Figure 6:**
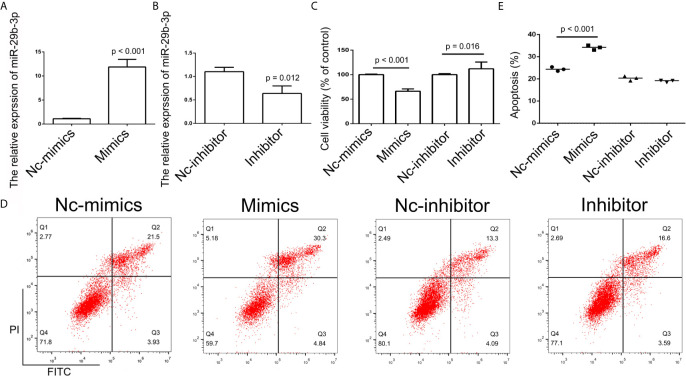
Analysis of miR-29b-3p expression. **(A, B)** Relative expression of miR-29b in the Nc-mimics, miR-29b-3p-mimics, Nc-inhibitor, and miR-29b-3p-inhibitor groups, utilizing qPCR in SCC9 cells. Data are represented as mean ± SD (n = 3). **(C)** Cell growth was assessed by the CCK-8 assay in SCC9 cells to determine the effect of miR-29b-3p on cell proliferation. **(D)** Cell apoptosis was analyzed after transfection with miR-29b-3p-mimics and miR29b-3p-inhibitor in SCC9 cells to determine the effect of miR-29b-3p on cell apoptosis. **(E)** Statistical analysis of the percentage of cell apoptosis among the four groups. Statistical significances between groups were evaluated using t-test for independent groups.

**Figure 7:**
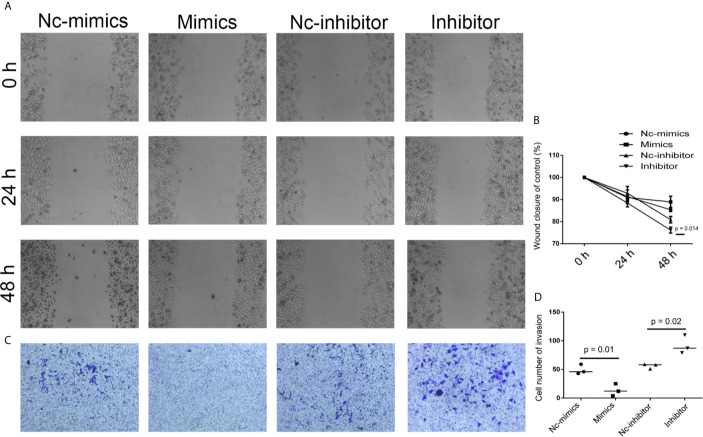
Analysis of cell migration and invasion after miR-29b knockdown and overexpression. **(A)** To determine the effect of miR-29b-3p on cell migration, cell migration was analyzed in Nc-mimics, miR-29b-3p-mimics, Nc-inhibitor, and miR-29b-3p-inhibitor groups, respectively, in SCC9 cells. **(B)** Statistical analysis of the percentage of cell migration among the four groups. **(C)** To determine the effect of miR-29b-3p on cell invasion, Cell invasion was analyzed in Nc-mimics, miR-29b-3p-mimics, Nc-inhibitor, and miR-29b-3p-inhibitor groups, respectively, in SCC9 cells. **(D)** Statistical analysis of the percentage of cell invasion among the four groups. Statistical significances between groups were evaluated using t-test for independent groups.

In order to further determine the effect of *XIST* on cell apoptosis, *XIST* was knocked down, as well as overexpressed, in SCC9 cells. As expected, the qPCR results demonstrated that *XIST* expression was inhibited in the si-XIST group, and overexpressed after the pcDNA3.1-XIST transfection (**Figures 4A, B**). The CCK-8 assay demonstrated that reduced expression of *XIST* led to an inhibition in the growth of SCC9 cells (**Figure 4C**) and induced apoptosis (**Figures 4D, E**). Next, we analyzed the effect of *XIST* expression on cell migration and invasion in SCC9 cells. The data indicated that overexpression of *XIST* is able to stimulate migration (**Figures 5A, B**) and invasion (**Figures 5C, D**), compared to the Con group. These results demonstrated that expression of *XIST* plays a role in cell growth, apoptosis, migration, and invasion. The role of miR-29b expression was also investigated in cell growth, apoptosis, migration, and invasion. The qPCR and CCK8 results revealed that increased expression of miR-29b led to inhibition of cell growth (**Figures 6A–C**) and induced cell apoptosis (**Figures 6D, E**). Considering that miR-29b positively regulates p53 protein, we investigated the expression of p53 (**Figure S4A**). Western blot and qPCR results validated that miR-29b mimics led to increased expression of p53 and E-cadherin (**Figure S4B, C**). In addition, increased expression of miR-29b led to inhibition of cell invasion (**Figures 7**, **S5**). In order to validate the role of miR-29b, a miR-29b inhibitor was transfected in the CuB group. The results demonstrated that miR-29b inhibitor promotes cell growth and inhibits apoptosis in SCC9 cells (**Figure S6**). Hence, our results validated that decreased expression of *XIST* induced apoptosis and suppressed cell growth through miR-29b in SCC9 cells.

### Anti-Tumor Effects of CuB *In Vivo*

**Figure 8:**
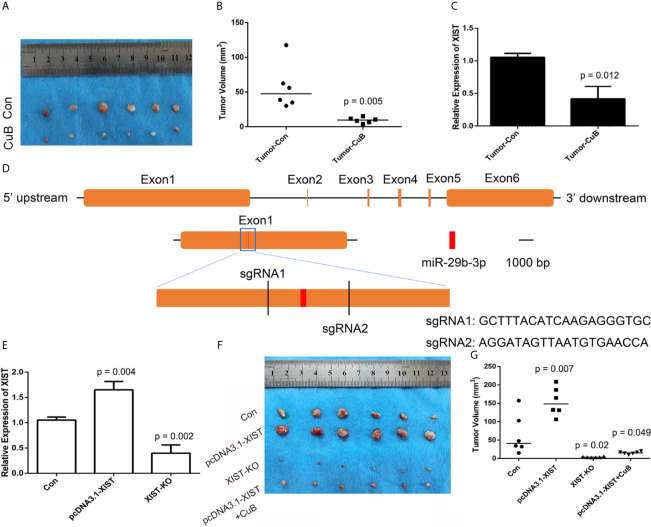
CRISPR/Cas9-mediated gene targeting of *XIST*. **(A)** Morphological observation of the mouse TSCC tumor tissue (N = 6 in each group). **(B)** Analysis of tumor volume. **(C)** Expression pattern of *XIST in* mice tumors after CuB treatment. Data are represented as mean ± SD (n = 3). **(D)** Schematic diagram of sgRNAs targeting the *XIST* gene loci. **(E)** Expression of *XIST* using qPCR in SCC9 cells. Data are represented as mean ± SD (n = 3). **(F)** Tumor morphology and **(G)** volume (N = 6 in each group). Statistical significances between groups were evaluated using t-test for independent groups. Yellow indicates *XIST*, while red indicates exon of miR-29b-3p.

In order to analyze CuB-mediated regulation of *XIST* expression in nude mice, SCC9 cells were injected. Compared to the Con group, the nude mice were treated with CuB (0.5 mg/kg) after 11 days. Our results demonstrated that CuB treatment significantly suppressed tumor growth *in vivo* (**Figures 8A, B**). The qPCR results indicated that CuB suppressed expression of *XIST* (**Figure 8C**). In order to analyze the effect of reduced expression of *XIST* in SCC9 cells, we designed two sgRNAs of *XIST* exon 1 (**Figure 8D**). The results demonstrated that *XIST* expression was lost in XIST KO cells (**Figure 8E**). Sanger sequence data validated the effect of gene editing of *XIST* (**Figure S7**). In order to investigate the *XIST* expression profile, XIST KO cells were injected into nude mice. Our *in vivo* data suggested that compared to the Con group, suppressed tumor growth occurred in the XIST KO group. Additionally, treatment with CuB in the *XIST* overexpression group suppressed tumor growth (**Figures 8F, G**). These results suggest that CuB has a function in the biological processes of cancer cells by regulating *XIST* expression.

## Discussion

CuB is a tetracyclic triterpene compound found in the *Cucurbitaceae* family, and has anti-inflammatory and anti-tumor effects on cancers. Considering that CuB has potential anti-cancer effects on breast cancer and CRC (17, 18), we analyzed cell invasion, migration, apoptosis and growth in TSCC cells that were treated with CuB. Our results demonstrated that CuB induced cell apoptosis and inhibited cell growth, migration and invasion in SCC9 cells. A previous study revealed that CuB inhibits cell growth by regulating expression of miR-146b-5p and the lncRNA *actin filament associated protein 1 antisense RNA1* in pancreatic cancer cells (6). In addition, CuB regulates the expression of the lncRNA *gastric cancer-associated transcript 3*, which induces apoptosis in gastric cancer cells (19). These results suggest that CuB may have a function in TSCC through a lncRNA-mediated mechanism.

Noncoding RNAs (ncRNAs) play significant roles in the development of various cancers. Specifically, lncRNAs are associated with being either tumor suppressors or oncogenes, and therefore, regulate the expression of genes and proteins in human cancer. In this study, RNA-seq revealed the presence of several differentially expressed lncRNAs that were associated with cell growth, apoptosis, migration and invasion after CuB treatment. Furthermore, the expression of *XIST* was found to be significantly reduced in both CAL27 and SCC9 cells. As an imprinted gene, *XIST* is crucial for embryo development and regulates XCI in mammals. However, *XIST* is abnormally expressed in various cancers, including hepatocellular carcinoma (HCC) (20). This indicates that the expression of *XIST* may be related to TSCC cancer development. A previous report demonstrated that reduced expression of *XIST* led to inhibition of tumor growth in thyroid cancer (21). Our results suggested that knockdown of *XIST* expression inhibited growth of SCC9 cells, which was validated from prior studies. Moreover, knockdown of *XIST* inhibited EMT in CRC (22). These data indicate that overexpression of *XIST* promotes cell migration and invasion. Furthermore, cellular apoptosis was induced by altered expression of *XIST* in HCC (23). The results of this study suggest that the expression of *XIST* has a function in cell apoptosis, growth, migration and invasion in TSCC.

Mounting evidence has revealed that miR-29b is a potential therapeutic candidate for CRC, HCC and lung cancer (24–26). In this study, bioinformatics analysis suggested that *XIST* has potentially competitive binding sites with miR-29b. Thus, miR-29b may play a role in TSCC. A previous report indicated that overexpression of miR-29b led to a reduction in myeloid cell leukemia 1 expression and induced cell apoptosis in KMCH cholangiocarcinoma cells (27). Additionally, miR-29b was targeted to the folate receptor 1 (*FOLR1*) in order to inhibit cell growth in CRC (28). Interestingly, previous RNA-seq data demonstrated that *FOLR1*, which has high expression in ovarian cancer, is differentially expressed in TSCC (29, 30). Indeed, miR-29b activates p53 protein, which induces cell apoptosis (13). Our results demonstrate that *XIST* regulates miR-29b expression, which induces cell apoptosis through the p53 pathway in TSCC cells.

In order to confirm the effect of *XIST* on TSCC *in vivo*, a mouse tumor model in nude mice was established. The *in vivo* data demonstrates suppressed expression of *XIST* after CuB treatment, which was further validated by our data in SCC9 cells. Moreover, CuB inhibited tumor growth in mice, which is in accordance with previous data in breast cancer (31). These results suggest that CuB is able to regulate the expression of *XIST*, which inhibits tumor development. In order to further investigate the putative function of *XIST* and miR-29b in TSCC, we analyzed KO and overexpressed *XIST*. The CRISPR/Cas9 system is powerful for gene editing in HCC and GC cells (32, 33). Our study revealed that *XIST* KO *via* the CRISPR/Cas9 system is able to inhibit tumor growth. These results suggest that reduced the expression of *XIST* inhibits tumor growth.

In summary, we identified 177 differentially expressed lncRNAs in TSCC cells after CuB treatment. These results suggested that expression of *XIST* is regulated by CuB in TSCC. Furthermore, knockdown of *XIST* and overexpression of miR-29b led to an inhibition of cell growth and invasion, and induced apoptosis. Collectively, our results indicate that CuB plays a role in TSCC by regulating expression of *XIST* and miR-29b, which regulates the p53 protein.

## Data Availability Statement

The datasets presented in this study can be found in online repositories. The names of the repository/repositories and accession number(s) can be found in the article/**Supplementary Material**.

## Ethics Statement

The studies involving human participants were reviewed and approved by Ethics Committee of the Hospital of Stomatology, Jilin University. The patients/participants provided their written informed consent to participate in this study. The animal study was reviewed and approved by Laboratory Animal Center of Jilin University. Written informed consent was obtained from the individual(s) for the publication of any potentially identifiable images or data included in this article.

## Author Contributions

DW and WL designed the experiments and wrote the manuscript. BT, SY, LC, ZY, and YL performed cell experiment and gene expression analysis. WL contributed reagents and materials. BT and HW carried out animal experiment. DW analyzed the data and prepared figures. All authors contributed to the article and approved the submitted version.

## Funding

Fundamental Research Funds for the Central Universities (no. 2019JCKT-70, 2020JCXK-45); Jilin Province Development and Reform Commission (no. 2019C051-5); Department of Finance of Jilin Province (no. JCSZ2019378-8); Jilin Scientific and Technological Development Program (no. 20190103071JH); Education Department of Jilin Province (no. JJKH20200950KJ).

## Conflict of Interest

The authors declare that the research was conducted in the absence of any commercial or financial relationships that could be construed as a potential conflict of interest.

## Supplementary Material

Supplementary Figure 1 Screen of cell growth related genes by RNA-seq. **(A)** LncRNAs were identified utilizing the CPC2, CNCI, Pfam and PLEK software. **(B)** LncRNAs on each chromosome. **(C)** The log2 fold change of lncRNAs.

Supplementary Figure 2 Analysis of *XIST* and miR-29b in TCGA data base. **(A)** The effect of *XIST* expression on survival of HNSCC patients, as per analysis of the TCGA data. **(B)** Effect of miR-29 expression on HNSCC patient survival, according to analysis of TCGA data.

Supplementary Figure 3 Screen of miRNA which was regulted by *XIST.*

Supplementary Figure 4 Analysis the expression pattern of p53 and E-cadherin in SCC9 cells. **(A)** Schematic diagram of XIST and miR-29b regulated p53 expression. **(B)** The expression of p53 and E-cadherin using Western blot. (C) The relative expression of TP53 and CDH1 using qPCR. Data are represented as mean ± SD (n = 3). Statistical significances between groups were evaluated using t-test for independent groups.

Supplementary Figure 5 Analysis of cell invasion by colony formation assay. **(A, B)** Cell invasion was analyzed using colony formation assay after CuB treatment in SCC9 cells **(C, D)** Cell invasion was analyzed after transfection with pcDNA3.1-XIST and si-XIST in SCC9 cells. **(E, F)** Cell invasion was analyzed after transfection with miR-29b-3p-mimics and miR29b-3p-inhibitor in SCC9 cells. Statistical significances between groups were evaluated using t-test for independent groups.

Supplementary Figure 6 Analysis of cell growth and apoptosis after transfection of miR-29b inhibitor in CuB-treated cells. **(A)** Cell growth was analyzed using the CCK-8 assay after transfection with the miR-29b-3p-inhibitor in CuB-treated (50 nM) SCC9 cells. **(B)** Cell apoptosis was assessed after miR-29b-3p-inhibitor transfection in CuB-treated (50 nM) SCC9 cells. **(C)** Statistical analysis of the percentage of cell apoptosis among the two groups. Statistical significances between groups were evaluated using t-test for independent groups.

Supplementary Figure 7 Analysis of XIST-KO by Sanger sequencing of SCC9 cells.
