# Supplementary figures and images for: Cucurbitacin B Inhibits Cell Proliferation by Regulating X-Inactive Specific Transcript Expression in Tongue Cancer
Source: Front Oncol. 2021 Jul 6;11:651648. doi: 10.3389/fonc.2021.651648 (PMC8290325; doi:10.3389/fonc.2021.651648)

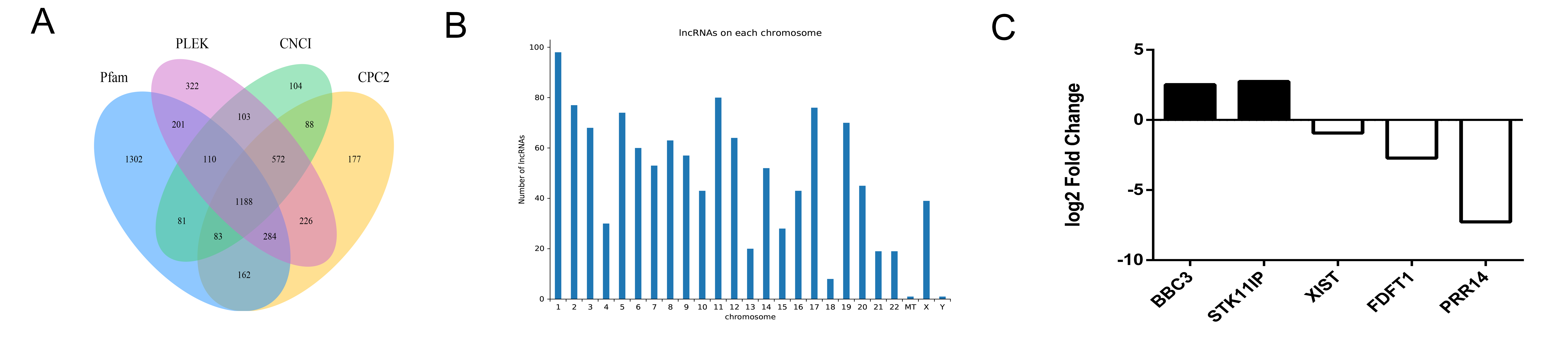

Supplement: Supplementary Figure 1 — Screen of cell growth related genes by RNA-seq. (A) LncRNAs were identified utilizing the CPC2, CNCI, Pfam and PLEK software. (B) LncRNAs on each chromosome. (C) The log2 fold change of lncRNAs. [file Image_1.tif]

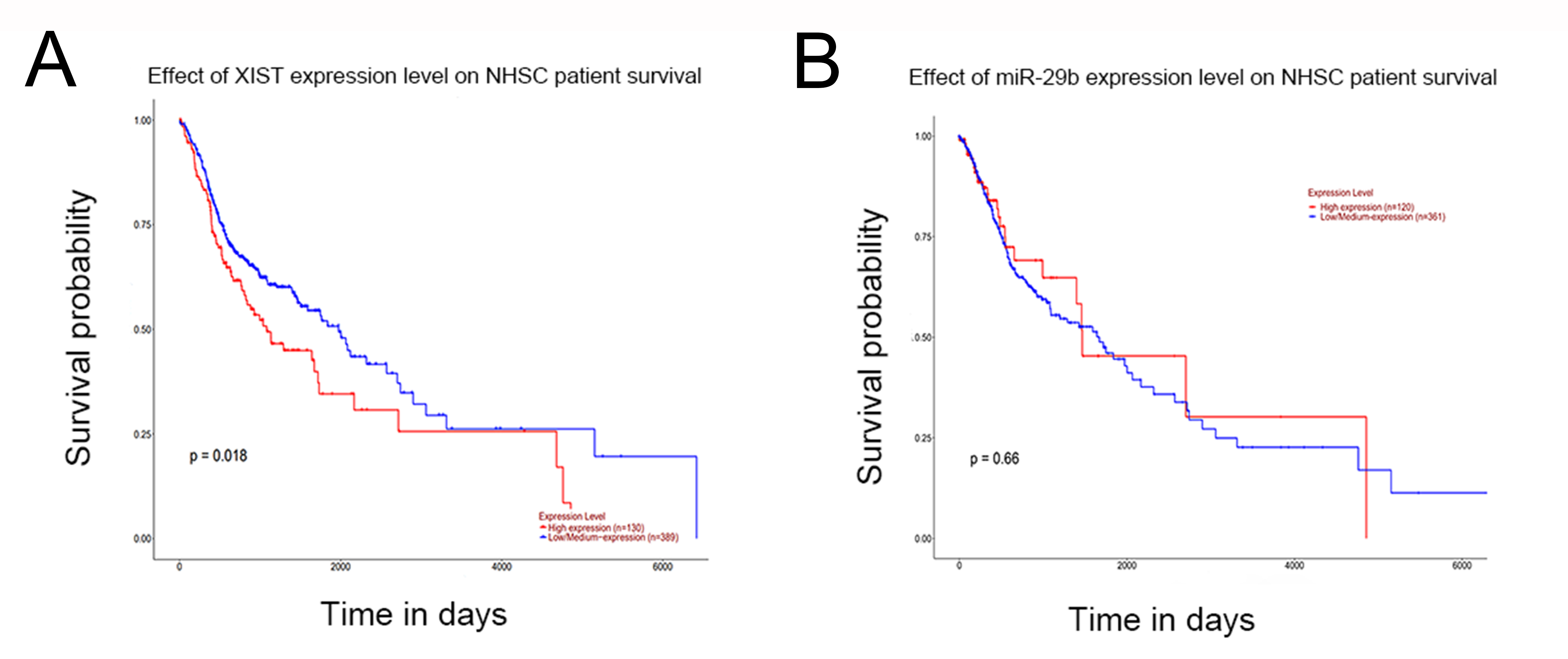

Supplement: Supplementary Figure 2 — Analysis of XIST and miR-29b in TCGA data base. (A) The effect of XIST expression on survival of HNSCC patients, as per analysis of the TCGA data. (B) Effect of miR-29 expression on HNSCC patient survival, according to analysis of TCGA data. [file Image_2.tif]

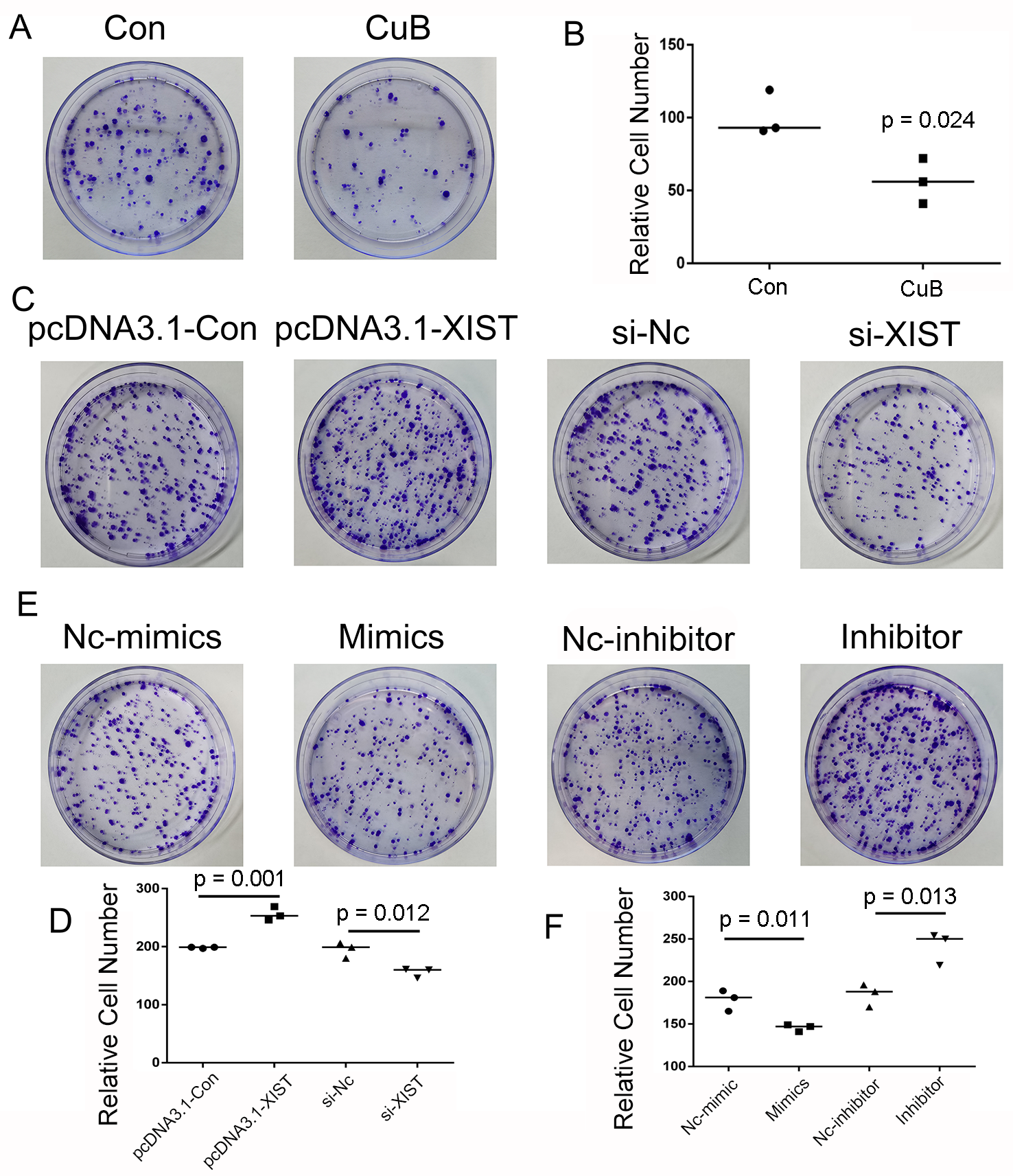

Supplement: Supplementary Figure 5 — Analysis of cell invasion by colony formation assay. (A, B) Cell invasion was analyzed using colony formation assay after CuB treatment in SCC9 cells (C, D) Cell invasion was analyzed after transfection with pcDNA3.1-XIST and si-XIST in SCC9 cells. (E, F) Cell invasion was analyzed after transfection with miR-29b-3p-mimics and miR29b-3p-inhibitor in SCC9 cells. Statistical significances between groups were evaluated using t-test for independent groups. [file Image_5.tif]

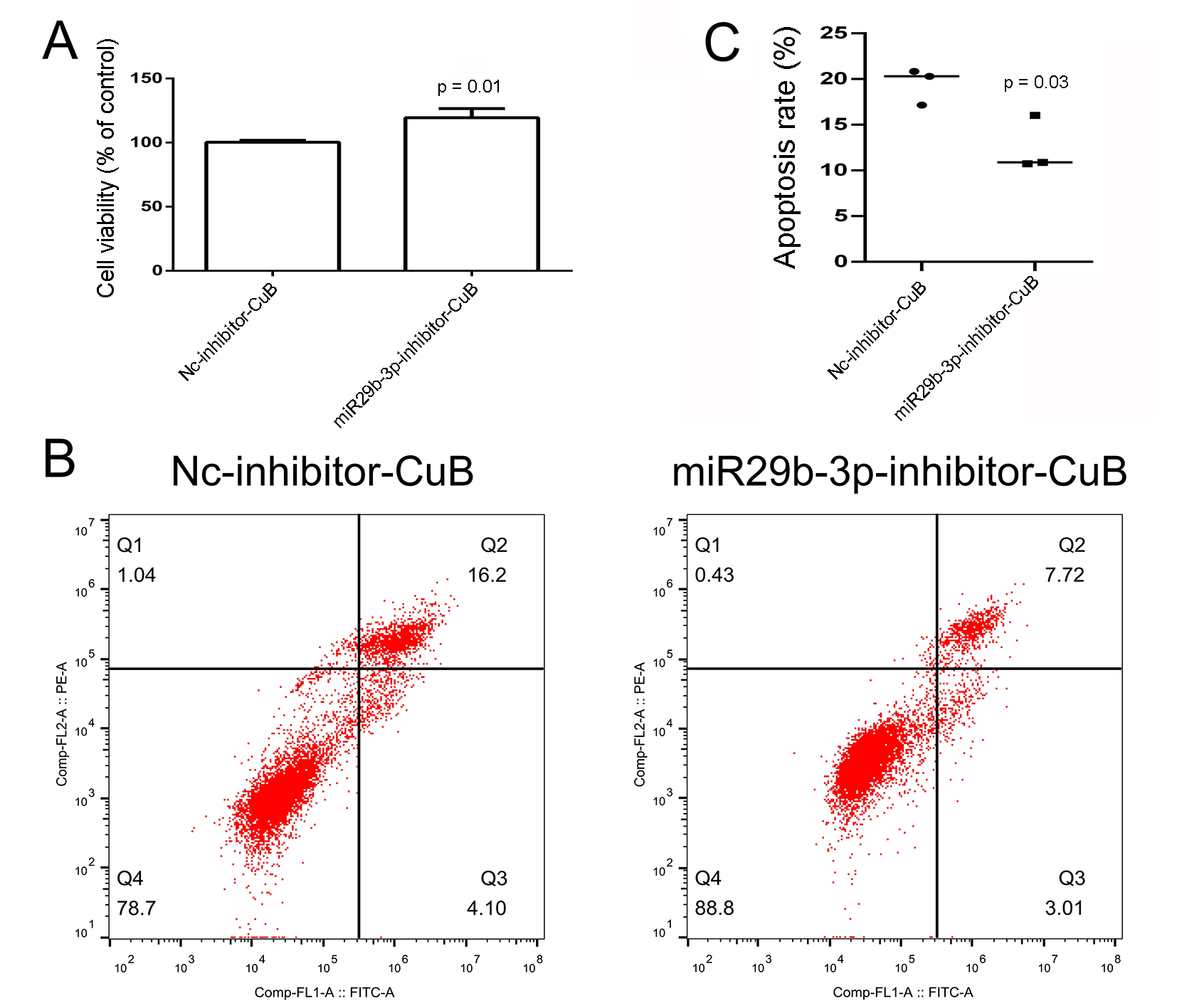

Supplement: Supplementary Figure 6 — Analysis of cell growth and apoptosis after transfection of miR-29b inhibitor in CuB-treated cells. (A) Cell growth was analyzed using the CCK-8 assay after transfection with the miR-29b-3p-inhibitor in CuB-treated (50 nM) SCC9 cells. (B) Cell apoptosis was assessed after miR-29b-3p-inhibitor transfection in CuB-treated (50 nM) SCC9 cells. (C) Statistical analysis of the percentage of cell apoptosis among the two groups. Statistical significances between groups were evaluated using t-test for independent groups. [file Image_6.tif]

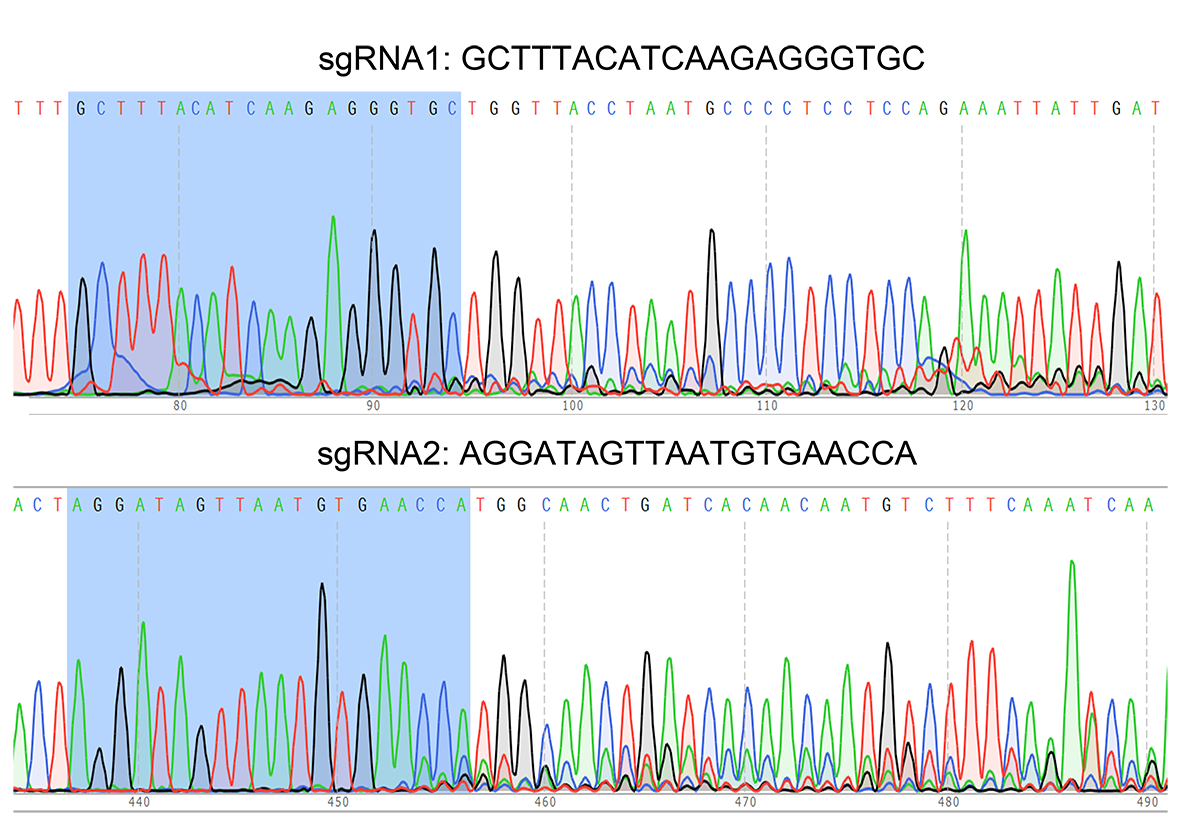

Supplement: Supplementary Figure 7 — Analysis of XIST-KO by Sanger sequencing of SCC9 cells. [file Image_7.tif]
