# Supplementary material for: Cucurbitacin B Inhibits Cell Proliferation by Regulating X-Inactive Specific Transcript Expression in Tongue Cancer
Source: Front Oncol. 2021 Jul 6;11:651648. doi: 10.3389/fonc.2021.651648 (PMC8290325; doi:10.3389/fonc.2021.651648)
Supplement: Supplementary file 9 [file Table_1.docx]

**Table**

Table S1 Sequence of sgRNA

| *SgRNA*s |  | | Sequences (5’→3’) | PAM | | |
| --- | --- | --- | --- | --- | --- | --- |
| SgRNA1 | |  | F: GCTTTACATCAAGAGGGTGC  R: GCACCCTCTTGATGTAAAGC | | TGG |  |
| SgRNA2 | |  | F: AGGATAGTTAATGTGAACCA  R: TGGTTCACATTAACTATCCT | | TGG |  |
| XIST  Identification | |  | F: GGGACCCTTACTACTCAA  R: ATAGAAAGGGATGCTGGA | |  |  |

Table S2 Primers for qPCR analysis

| Genes | Annealing (°C) | | Primer sequences (5’→3’) |
| --- | --- | --- | --- |
| *XIST* | | 60 | F: CCAACTGCTCACACCCTCTTA  R: AGGTCATCCACTGCTGAACA |
| *miR-29b-3p* | | 60 | F:ACACTCCAGCTGGGTAGCACCATTTGAAATC  R: TGGTGTCGTGGAGTCG  RT:CTCAACTGGTGTCGTGGAGTCGGCAATTCAGTTGAGAACACTGA |
| *STK11IP* | | 60 | F: CTTGTTGGTGTGTCCCCTG  R: GTGCGAGCTGCTTGGAGTT |
| *FDFT1* | | 60 | F: GCAACGCAGTGTGCATATTTT  R: CGCCAGTCTGGTTGGTAAAGG |
| *PRR14* | | 60 | F: GCACCACAGCTACCATCAGG  R: CCGGTCCACCTTTTGTGAAG |
| *BBC3* | | 60 | F: GACCTCAACGCACAGTACGAG  R: AGGAGTCCCATGATGAGATTGT |
| *U6* | | 60 | F:GCTTCGGCAGCACATATACTAAAAT  R: CGCTTCACGAATTTGCGTGTCAT |
| *GAPDH* | | 60 | F: TGGTATCGTGGAAGGACTCA  R: GGGCCATCGACAGTCTTC |

Table S3 Sequence of miR-29b-3p and *XIST* exon1

| Gene | sequence |
| --- | --- |
| miR-29b-3p  *XIST* exon1 | UAGCACCAUUUGAAAUCAGUGUU  GGGACCCTTACTACTCAAGACCTCTGTACTAGGACAGTTTATGTGCACAATCCTAATTGATTAGAACTGAGTCTTTTATATCAAGGTCCCTGCATCATCTTTGCTTTACATCAAGAGGGTGCTGGTTACCTAATGCCCCTCCTCCAGAAATTATTGATGTGCAAAATGCAATTTCCCTATCTGCTGTTAGTCTGGGGTCTCATCCCCTCATATTCCTTTTGTCTTACAGCAGGGGGTACTTGGGACTGTTAATGCGCATAATTGCAATTATGGTCTTTTCCATTAAATTAAGATCCCAACTGCTCACACCCTCTTAGCATTACAGTAGAGGGTGCTAATCACAAGGACATTTCTTTTGTACTGTTAATGTGCTACTTGCATTTGTCCCTCTTCCTGTGCACTAAAGACCCCACTCACTTCCCTAGTGTTCAGCAGTGGATGACCTCTAGTCAAGACCTTTGCACTAGGATAGTTAATGTGAACCATGGCAACTGATCACAACAATGTCTTTCAGATCAGATCCATTTTATCCTCCTTGTTTTACAGCAAGGGATATTAATTACCTATGTTACCTTTCCCTGGGACTATGAATGTGCAAAATTCCAATGTTCATGGTCTCTCCCTTTAAACCTATATTCTACCCCTTTTACATTATAGAAAGGGATGCTGGA |
