# Supplementary material for: Cucurbitacin B Inhibits Cell Proliferation by Regulating X-Inactive Specific Transcript Expression in Tongue Cancer
Source: Front Oncol. 2021 Jul 6;11:651648. doi: 10.3389/fonc.2021.651648 (PMC8290325; doi:10.3389/fonc.2021.651648)
Supplement: Supplementary file 8 [file DataSheet_1.zip › Data Sheet 1.ZIP!/apoptosis/con-Layout.pdf]

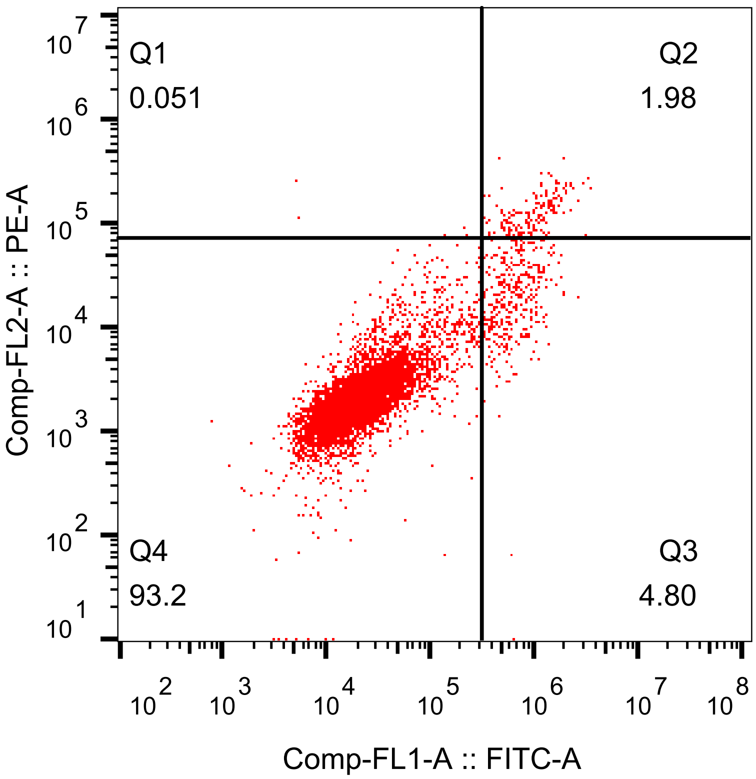

con-1

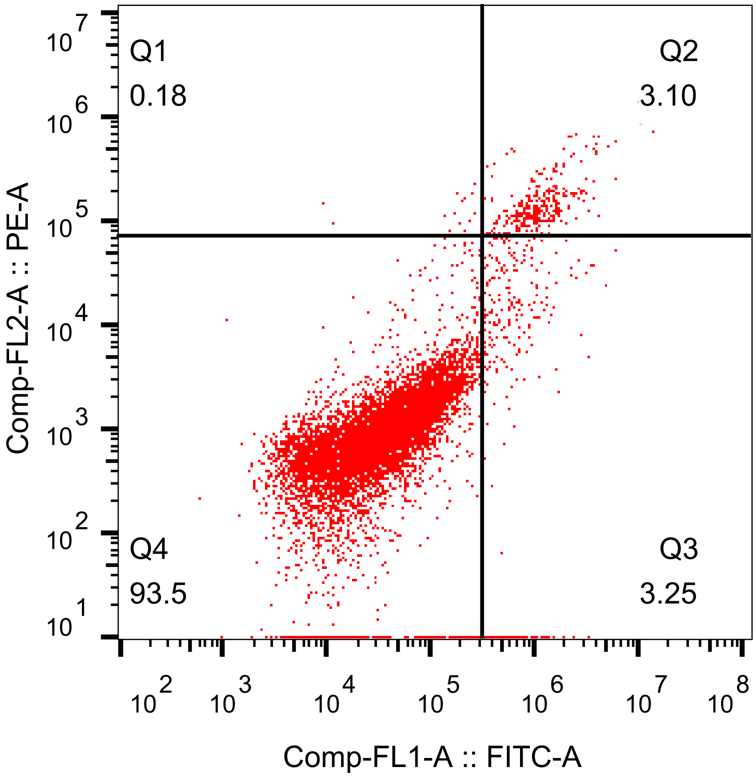

con-2

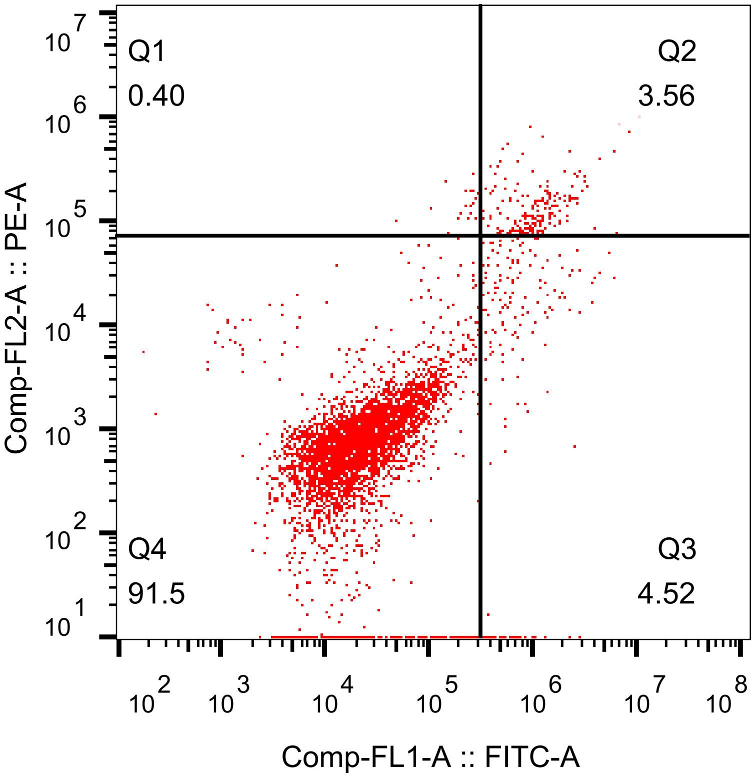
Q1
0.40
Q2
3.56
Q4
91.5
Q3
4.52
Comp-FL2-A :: PE-A
Comp-FL1-A :: FITC-A
10^1
10^2
10^3
10^4
10^5
10^6
10^7
10^8

con-3
