# Supplementary material for: Cucurbitacin B Inhibits Cell Proliferation by Regulating X-Inactive Specific Transcript Expression in Tongue Cancer
Source: Front Oncol. 2021 Jul 6;11:651648. doi: 10.3389/fonc.2021.651648 (PMC8290325; doi:10.3389/fonc.2021.651648)
Supplement: Supplementary file 8 [file DataSheet_1.zip › Data Sheet 5.ZIP!/fig6/apoptosis/inhibitor-Layout.pdf]

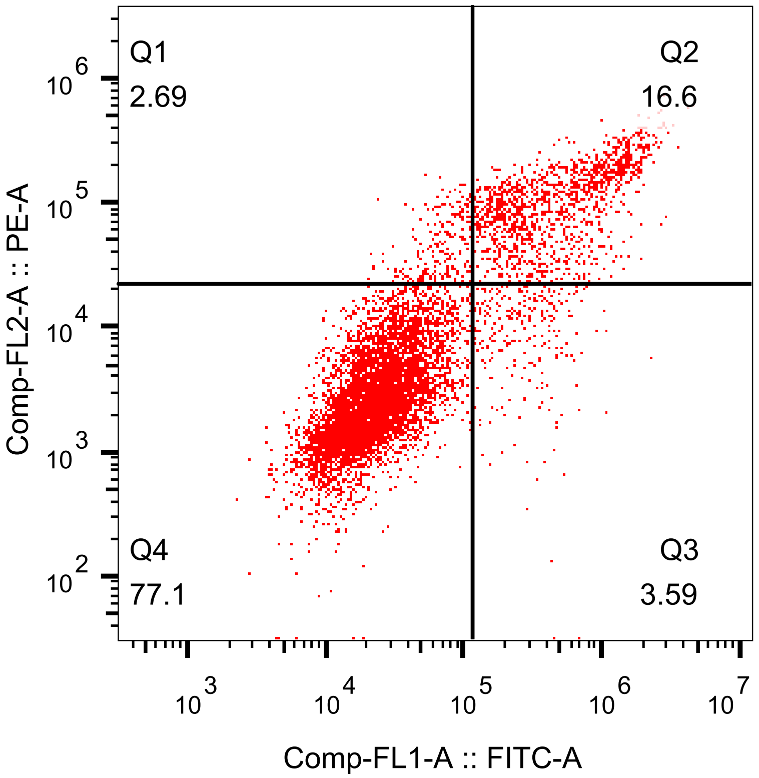
Q1
2.69
Q2
16.6
Q4
77.1
Q3
3.59
Comp-FL2-A :: PE-A
Comp-FL1-A :: FITC-A
10^2
10^3
10^4
10^5
10^6
10^7

inhibitor

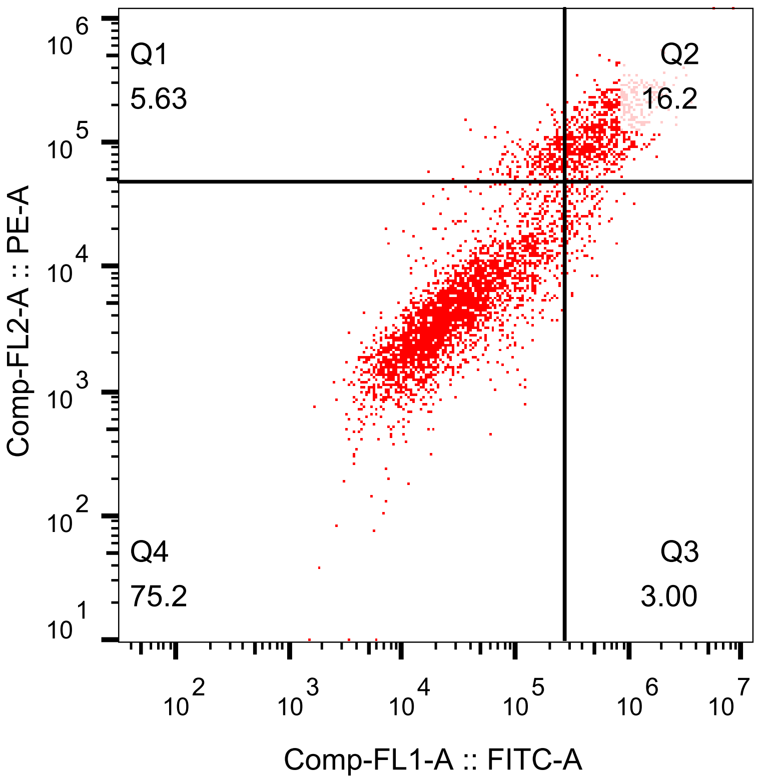

inhibitor

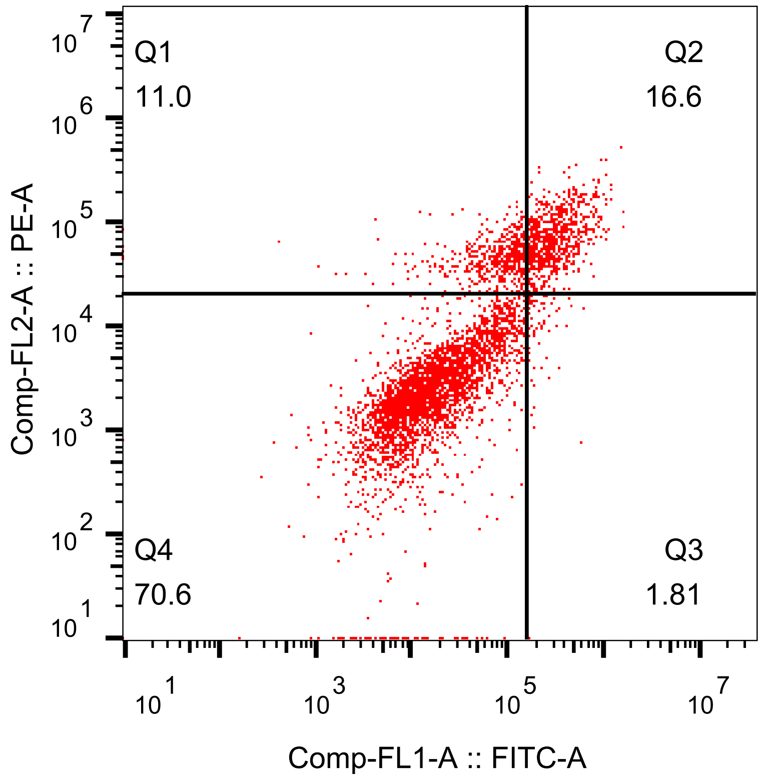

inhibitor
