# Supplementary material for: Cucurbitacin B Inhibits Cell Proliferation by Regulating X-Inactive Specific Transcript Expression in Tongue Cancer
Source: Front Oncol. 2021 Jul 6;11:651648. doi: 10.3389/fonc.2021.651648 (PMC8290325; doi:10.3389/fonc.2021.651648)
Supplement: Supplementary file 8 [file DataSheet_1.zip › Data Sheet 5.ZIP!/figS5/clone.pdf]

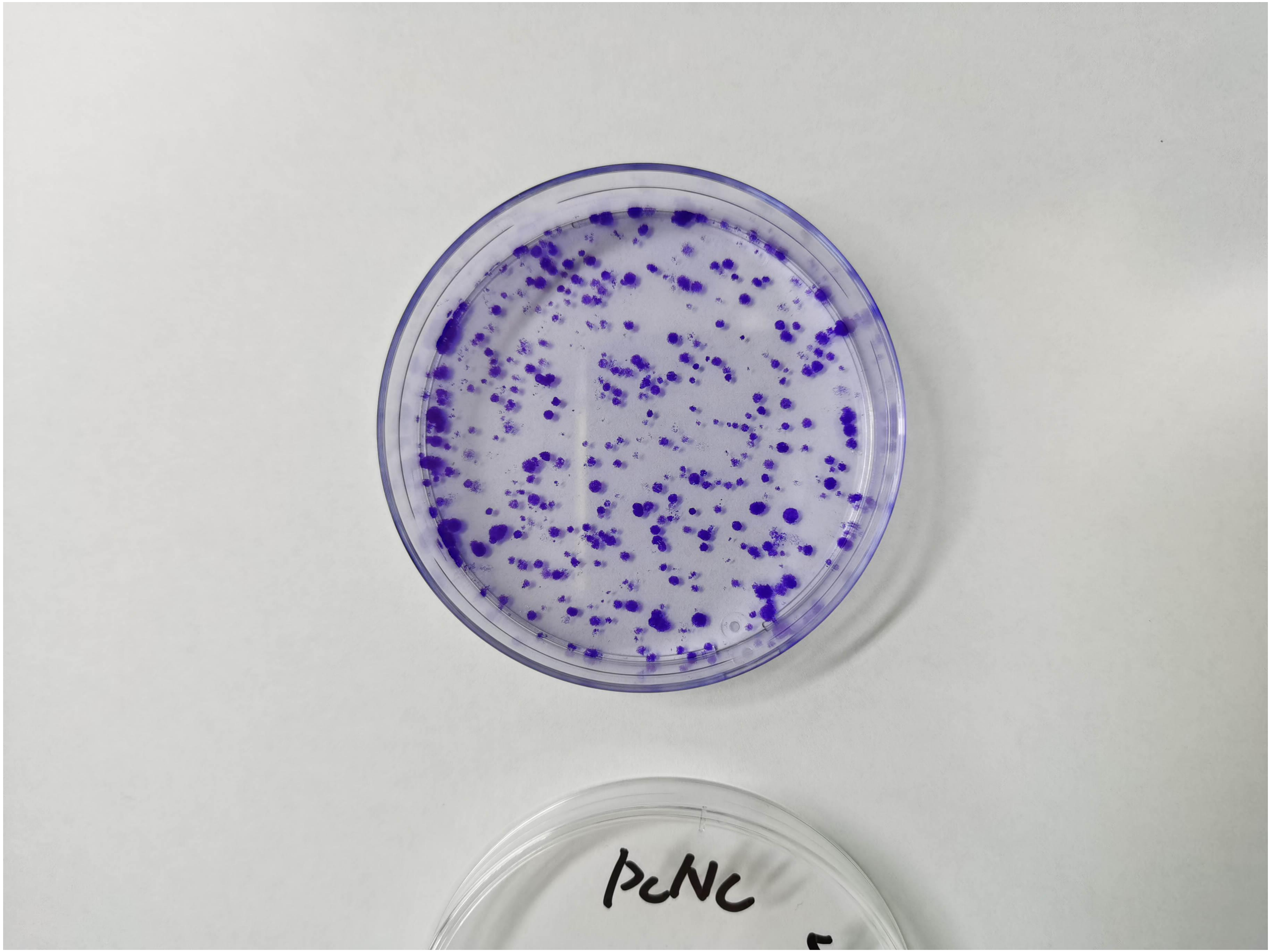
PcNC

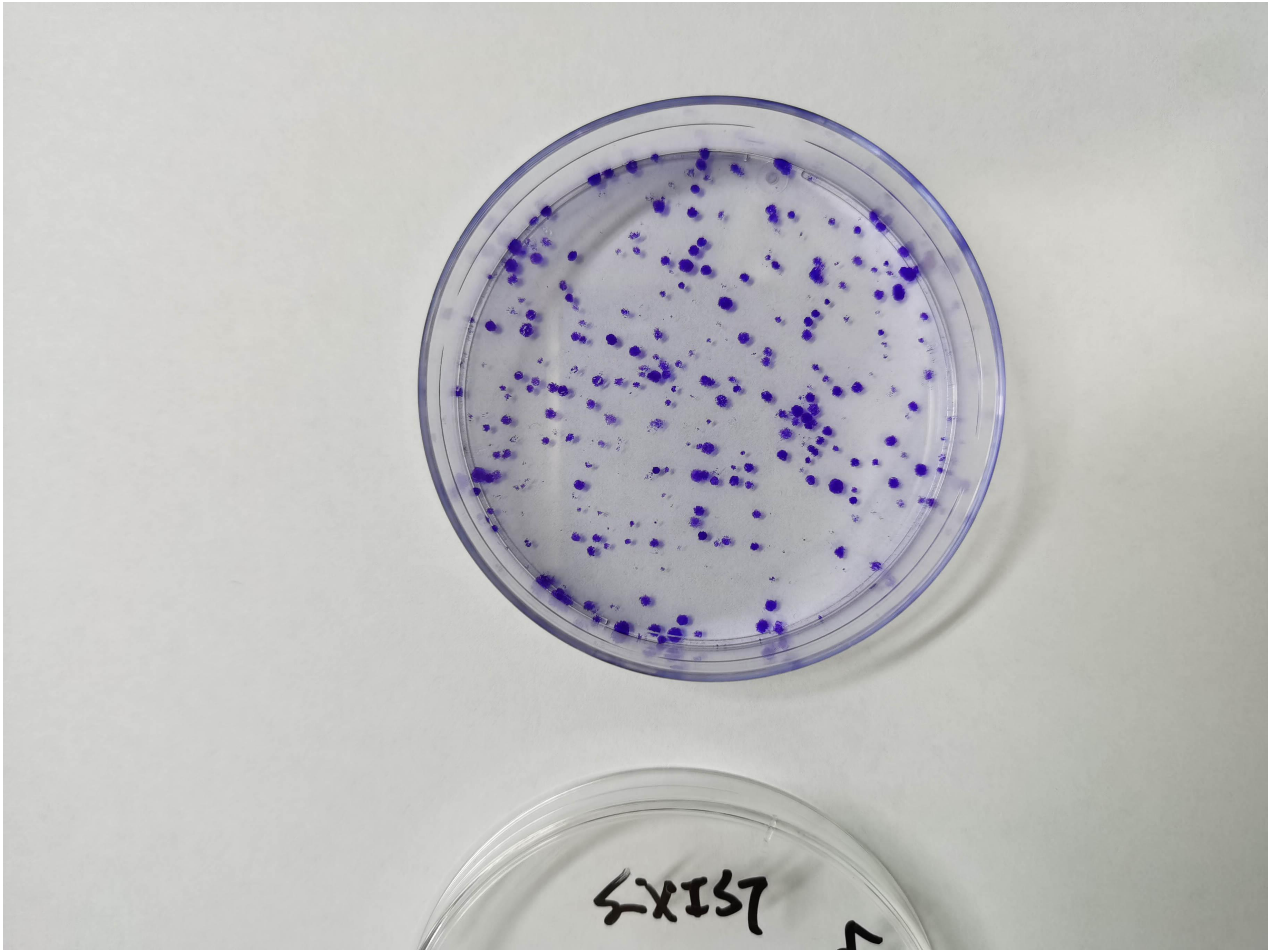

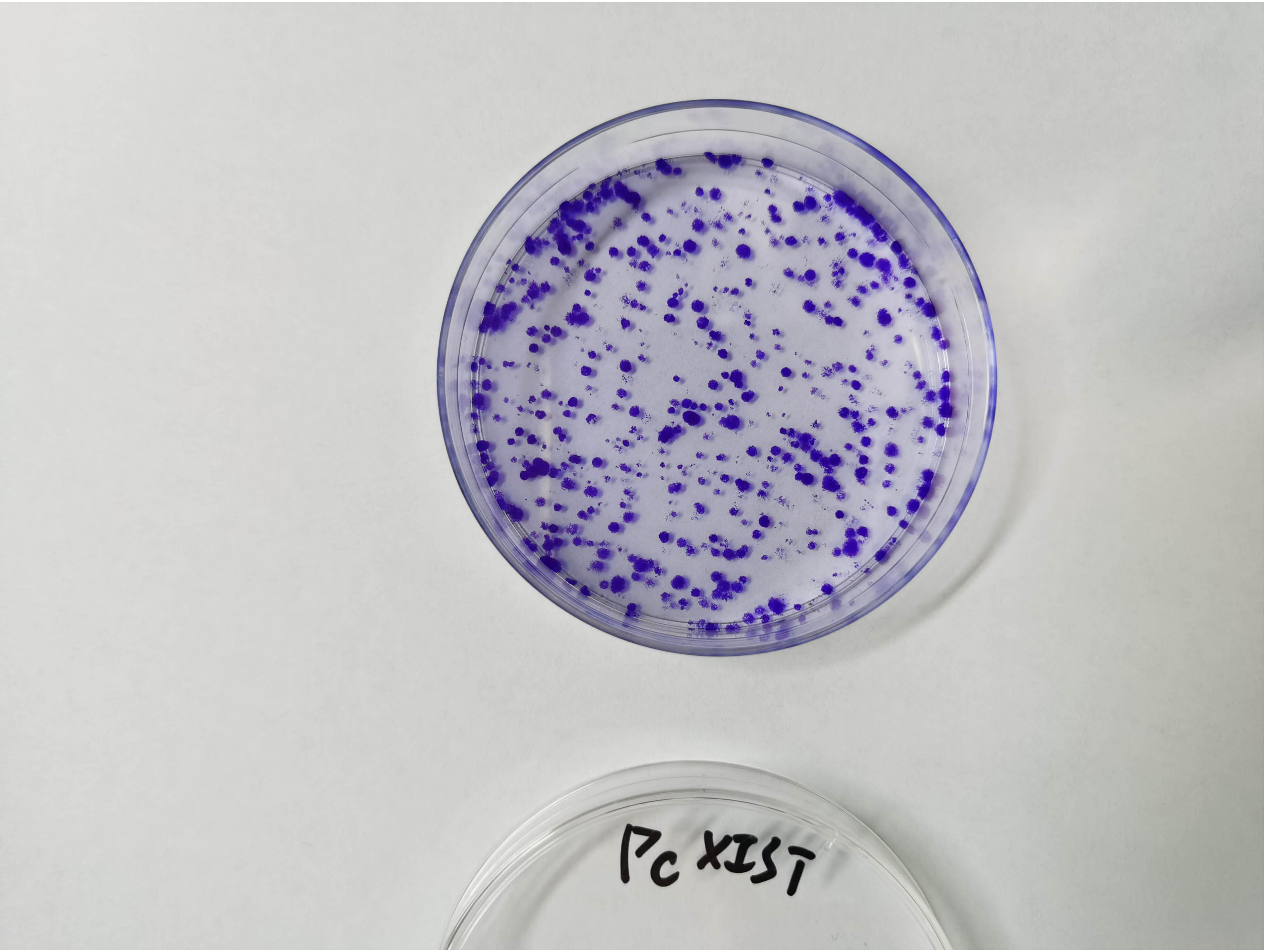
PC XIST

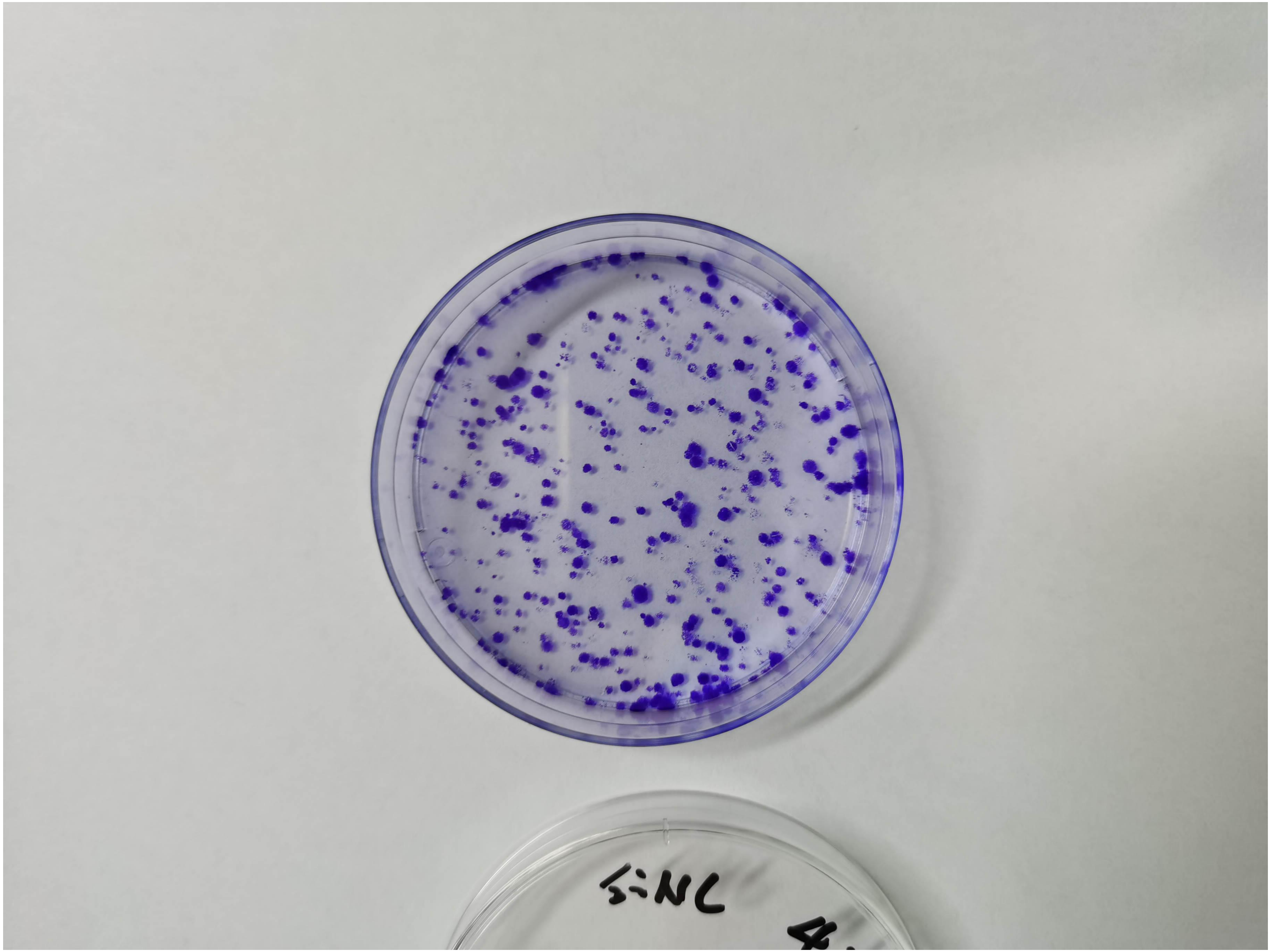
si-NC

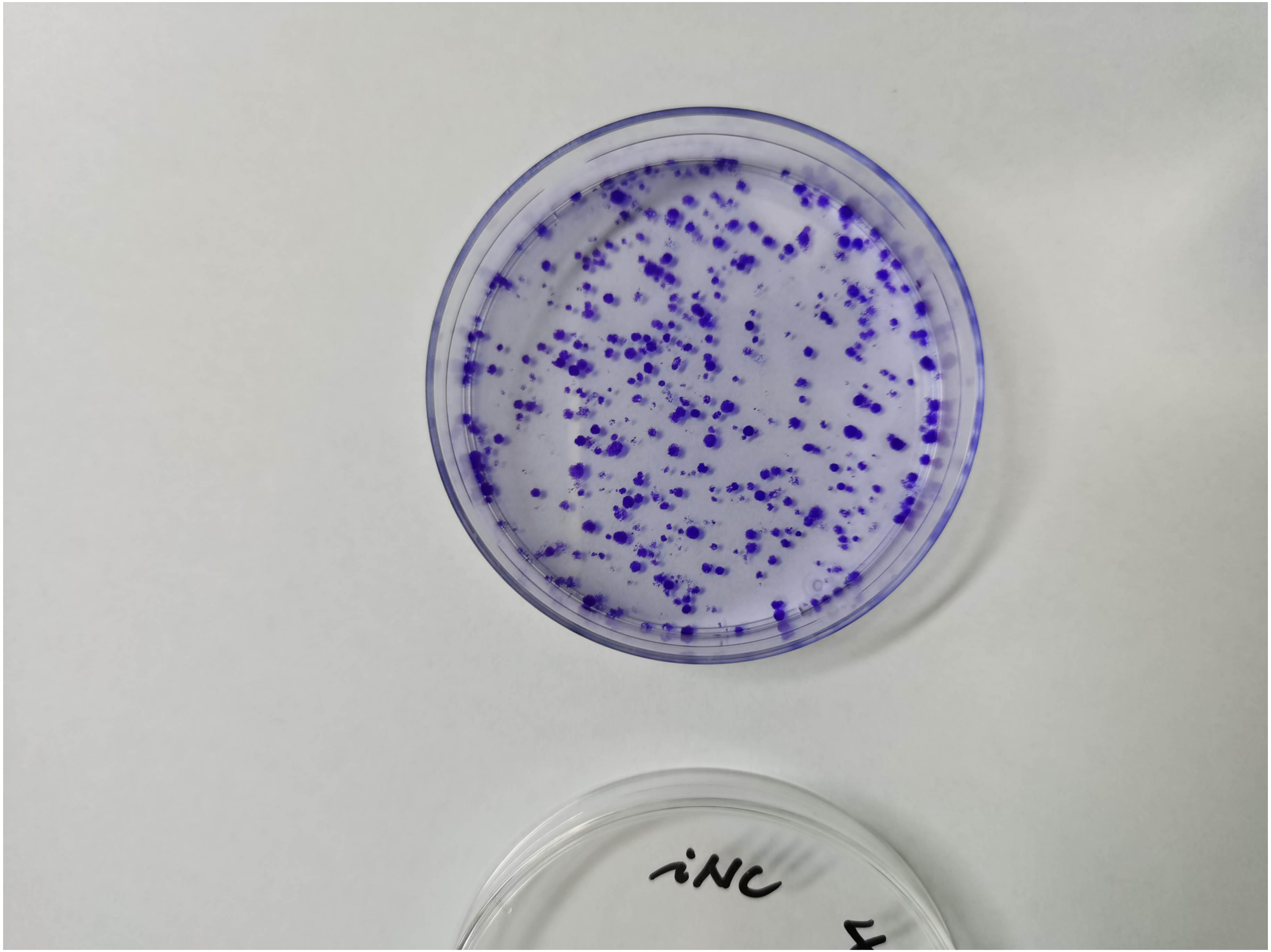
iNC

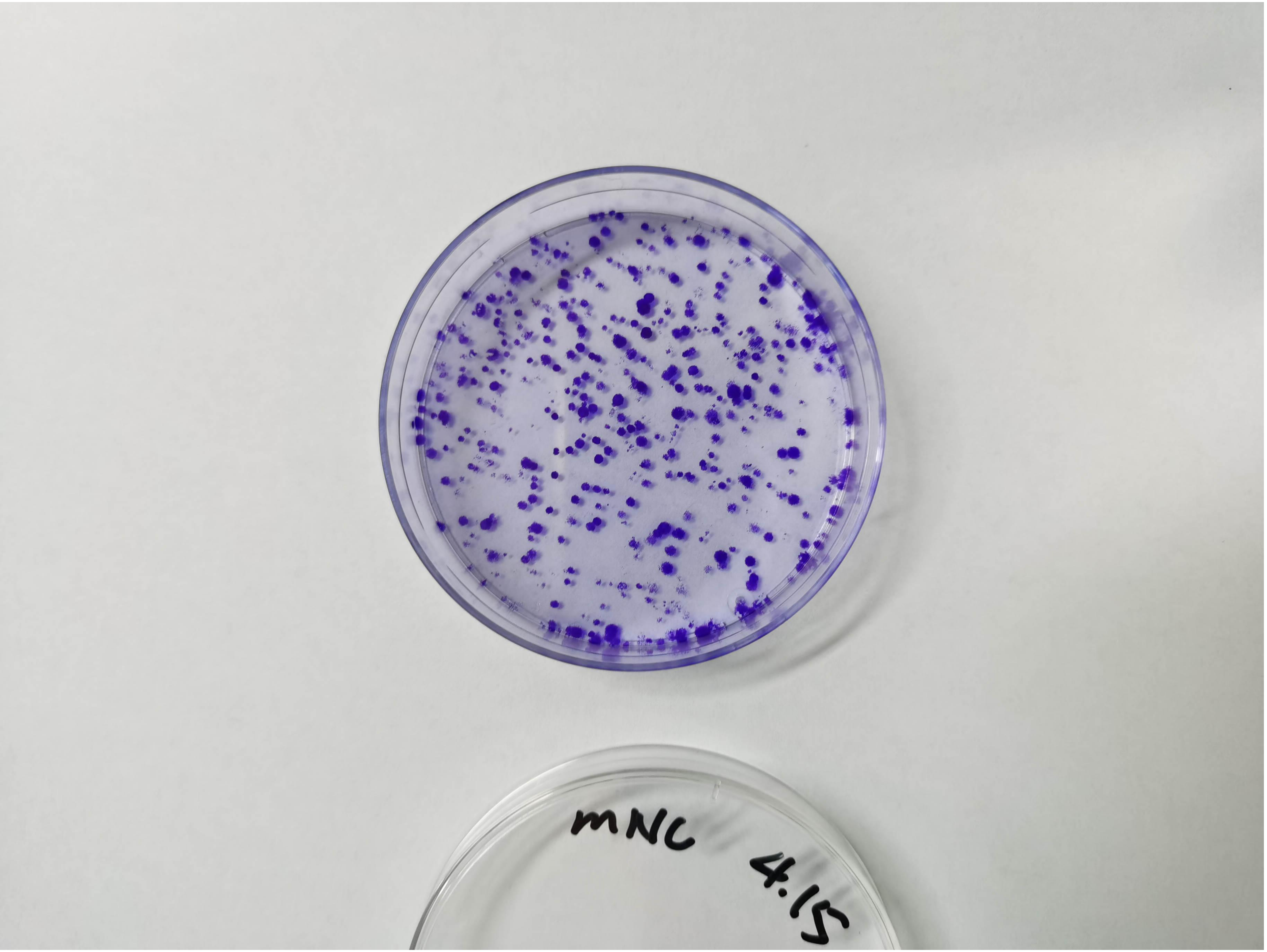
MNC
4.15

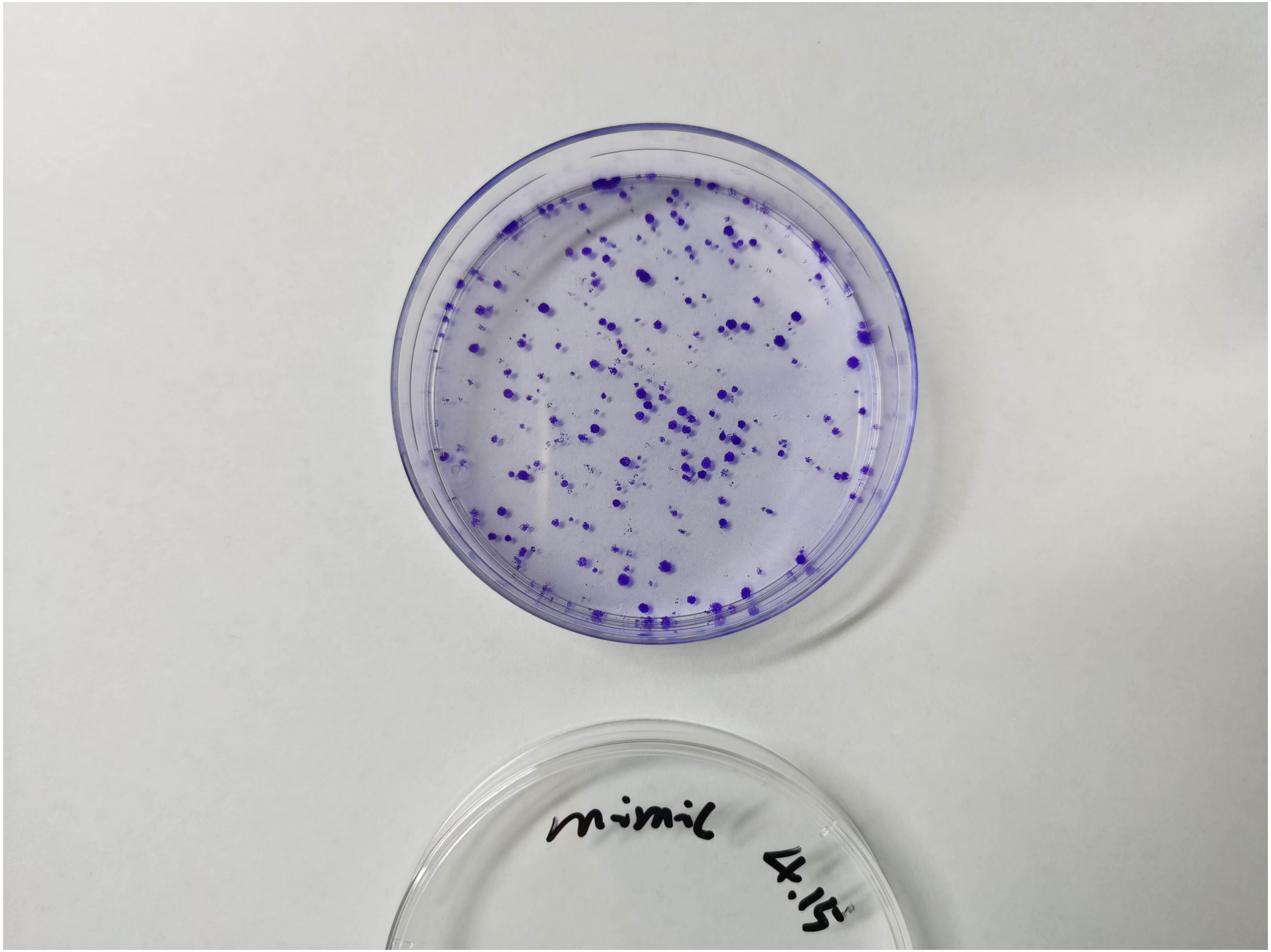
mimic
4.15

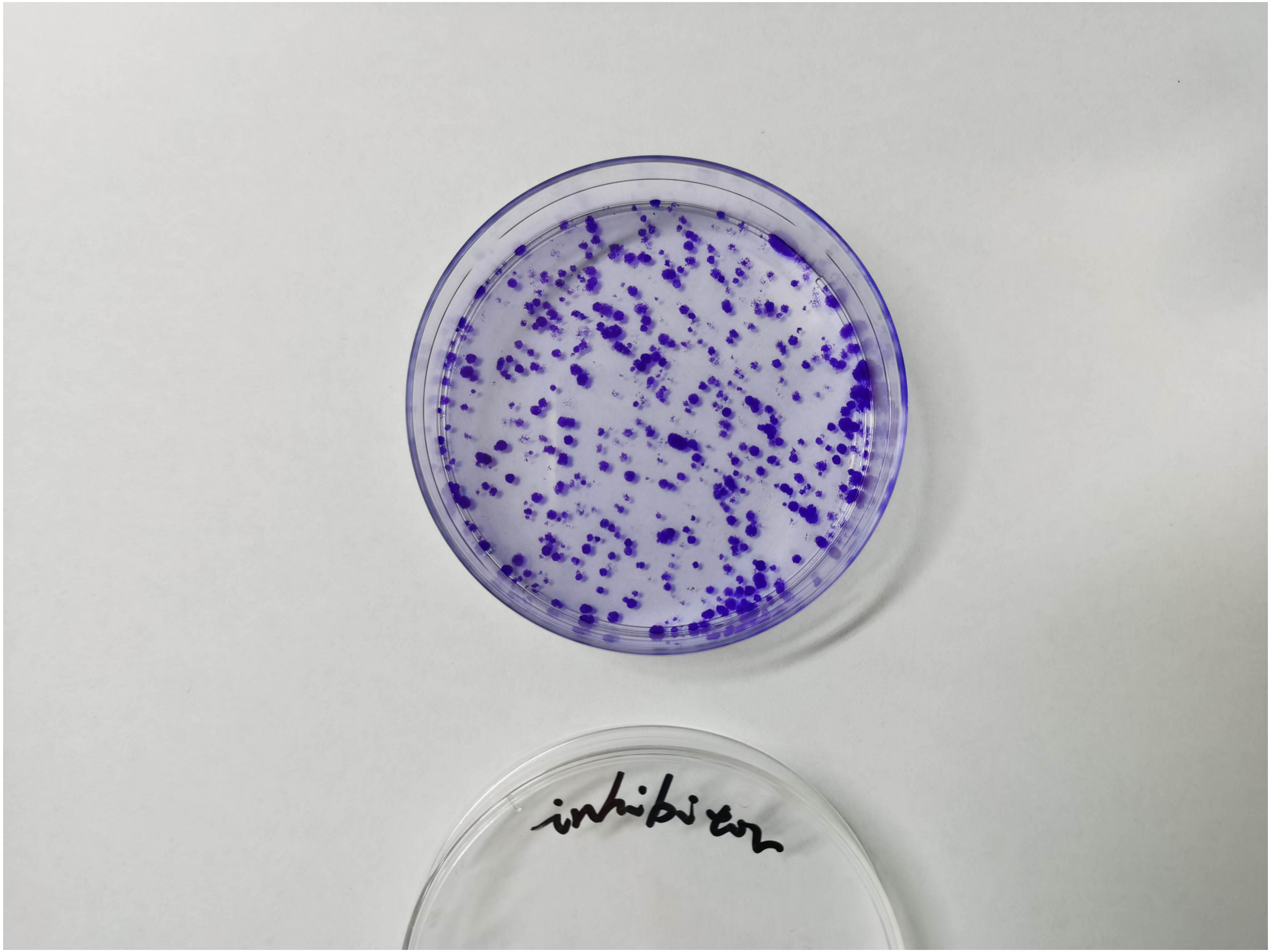
inhibitor

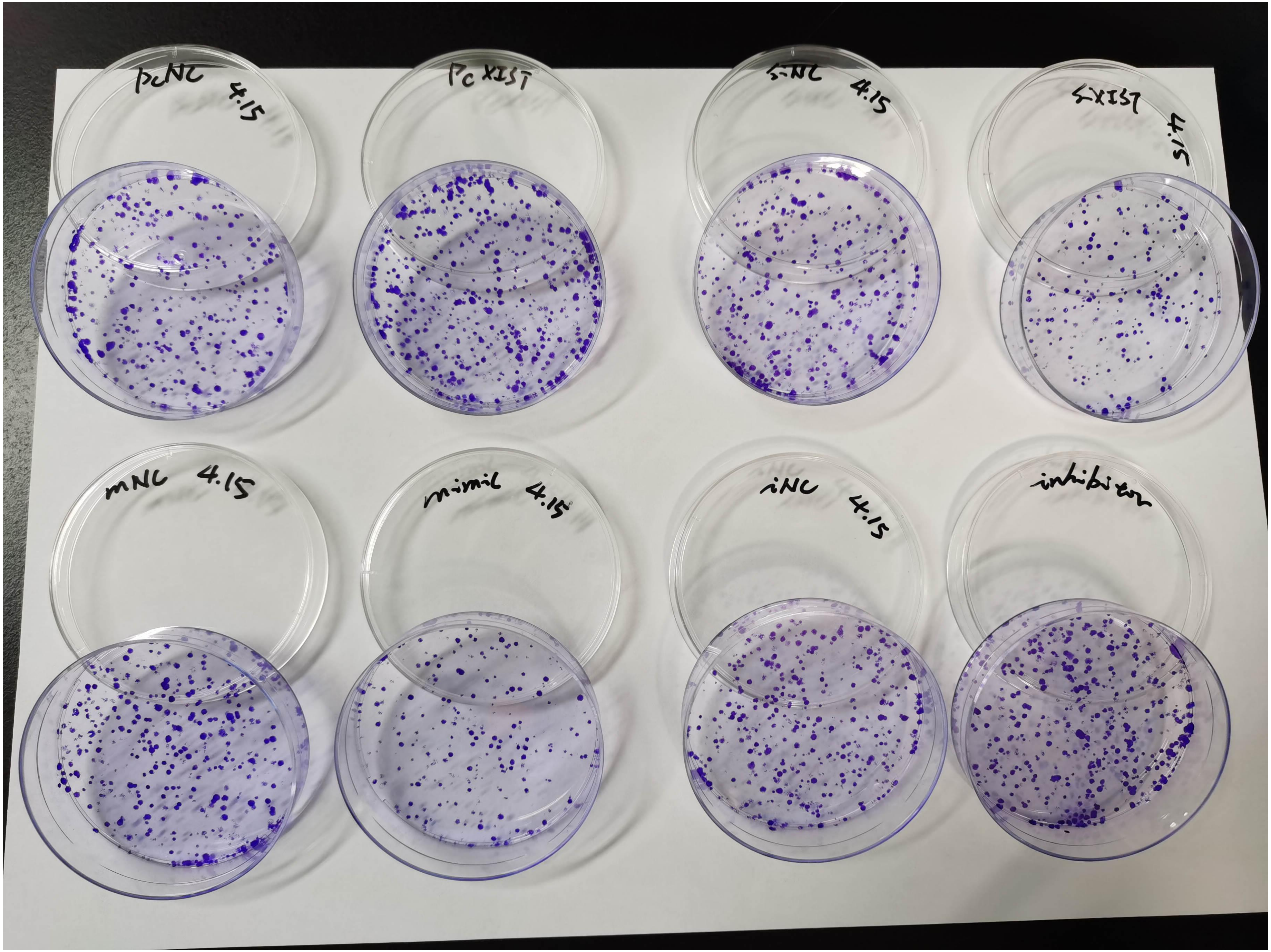
pcNC 4.15
pc XIST
si-NC 4.15
si-XIST 4.15
mNC 4.15
mimic 4.15
iNC 4.15
inhibitor

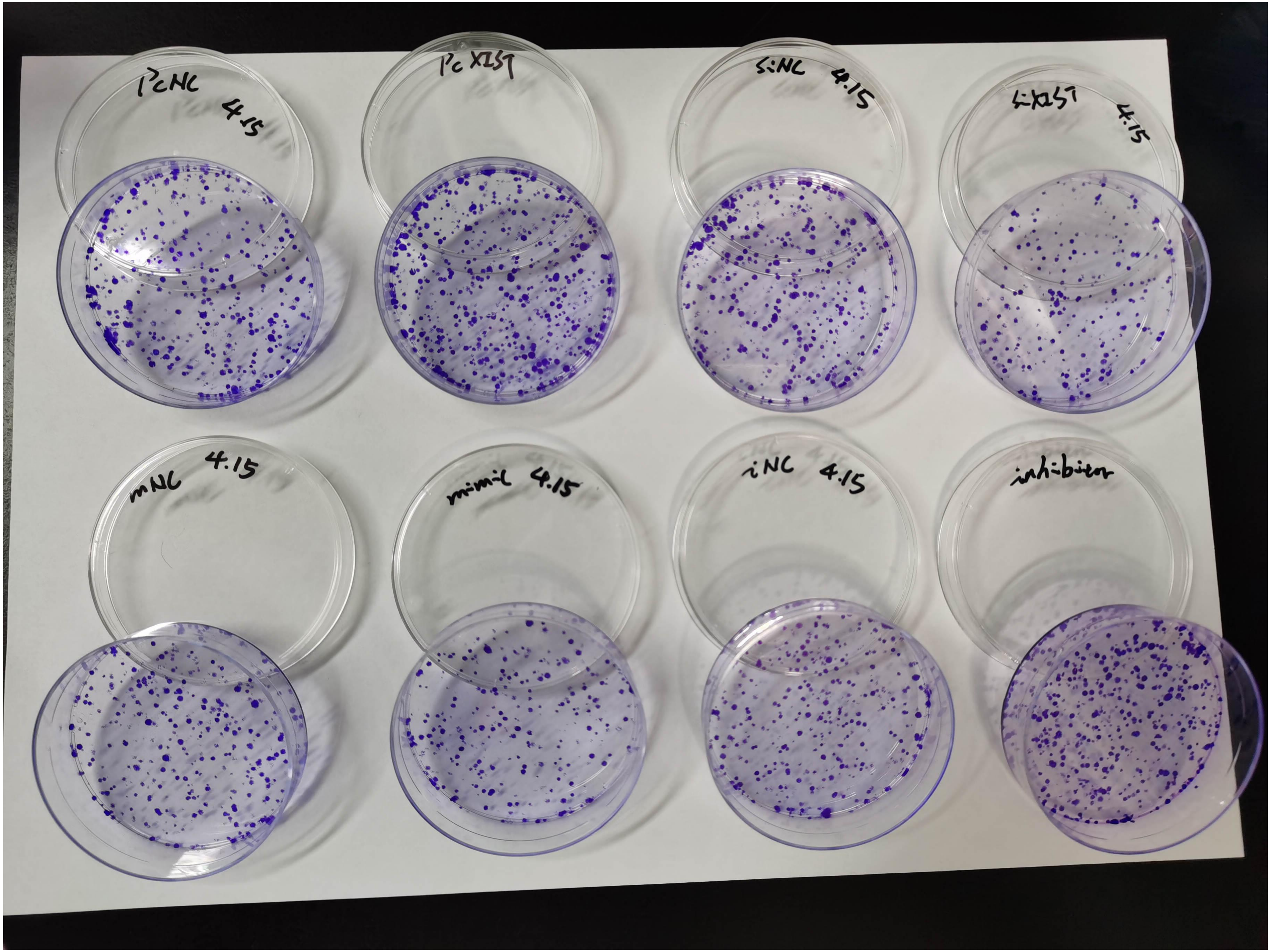
PcNC 4.15
Pc XIST
siNC 4.15
siXIST 4.15
mNC 4.15
mimic 4.15
iNC 4.15
inhibitor

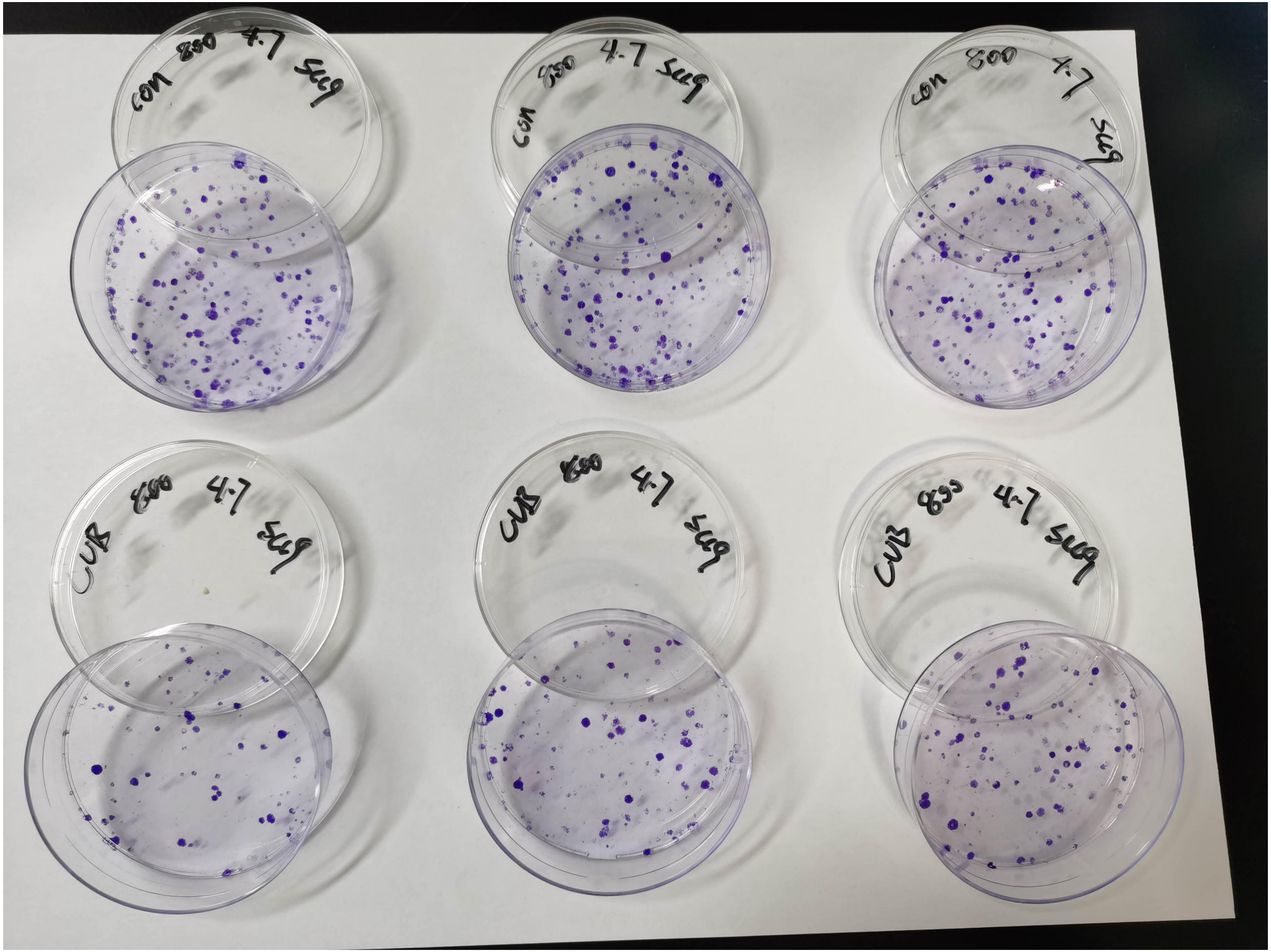
con 800 4.7 5ug
con 800 4.7 5ug
con 800 4.7 5ug
CUB 800 4.7 5ug
CUB 800 4.7 5ug
CUB 800 4.7 5ug

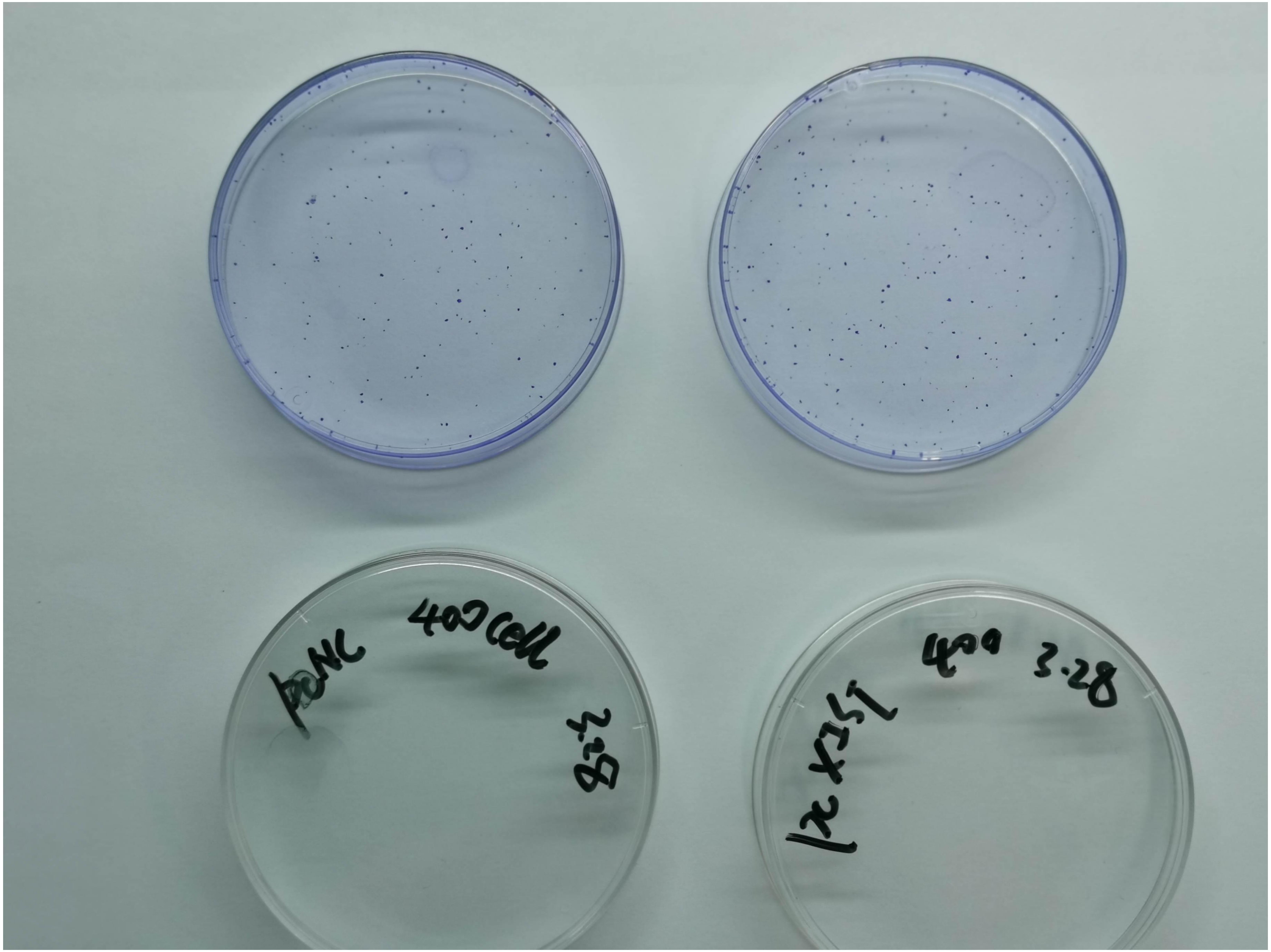

400 cell
400
3.28

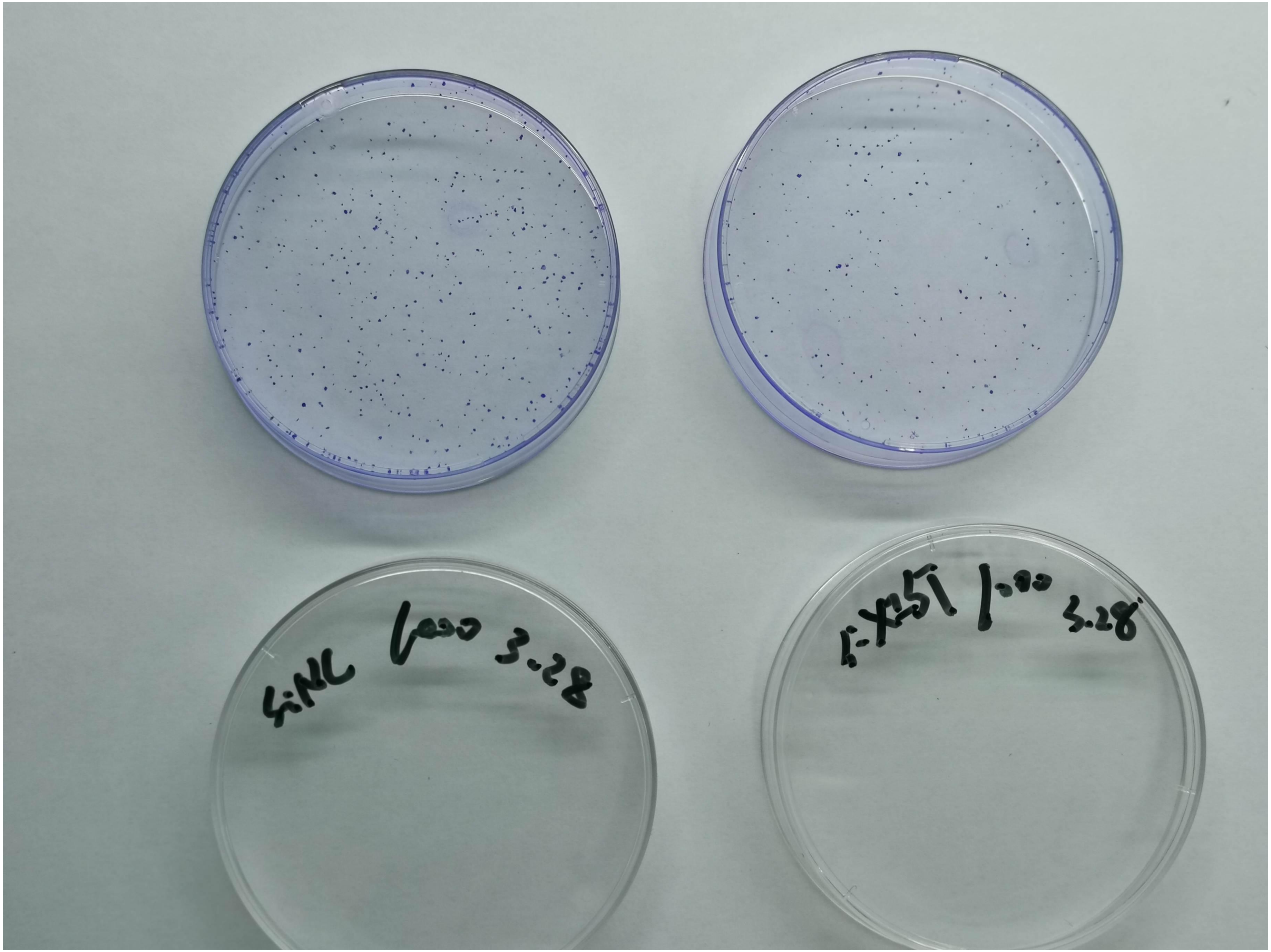
siNC 1000 3.28
3.28

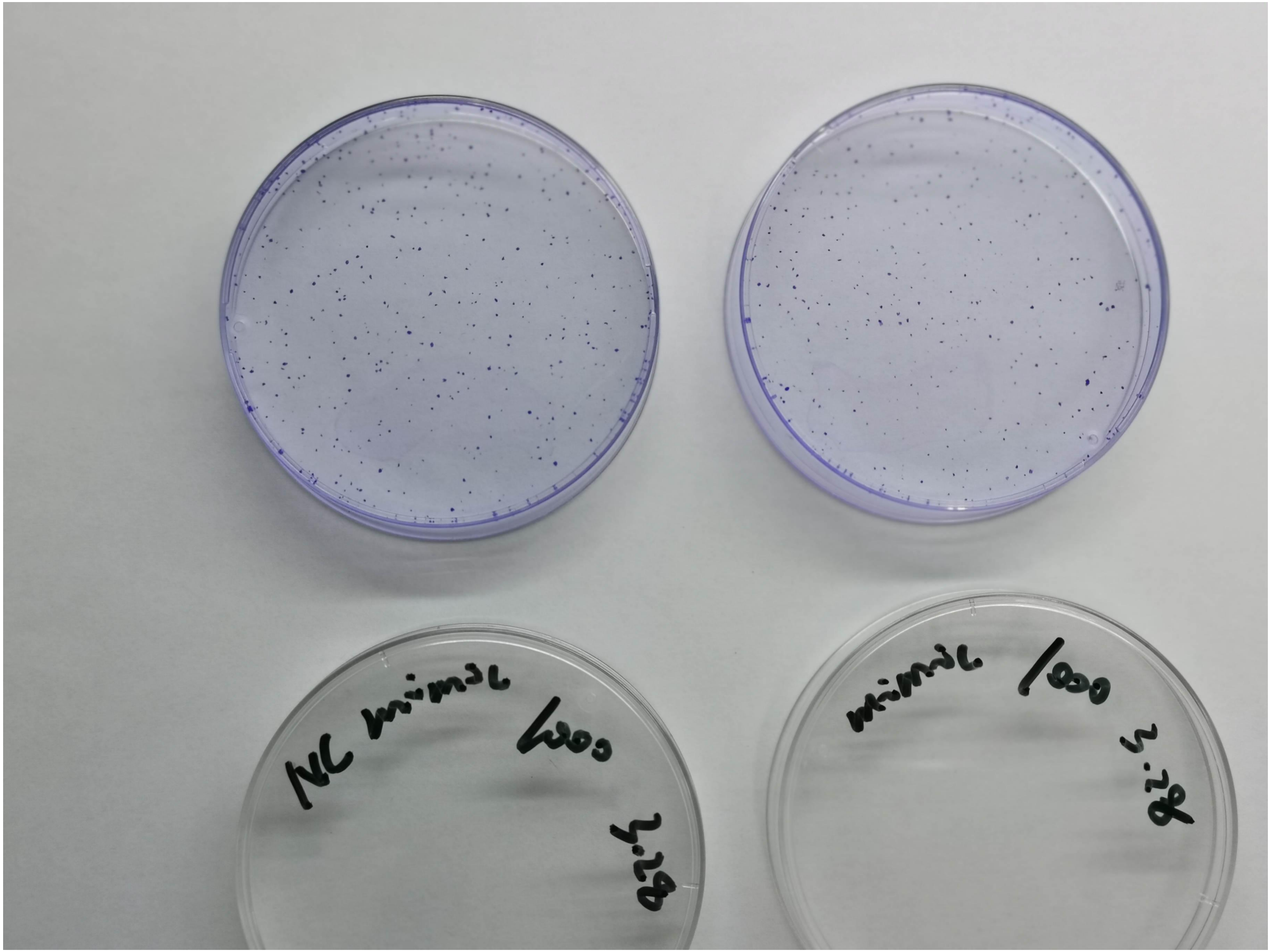
NC mimic
mimic

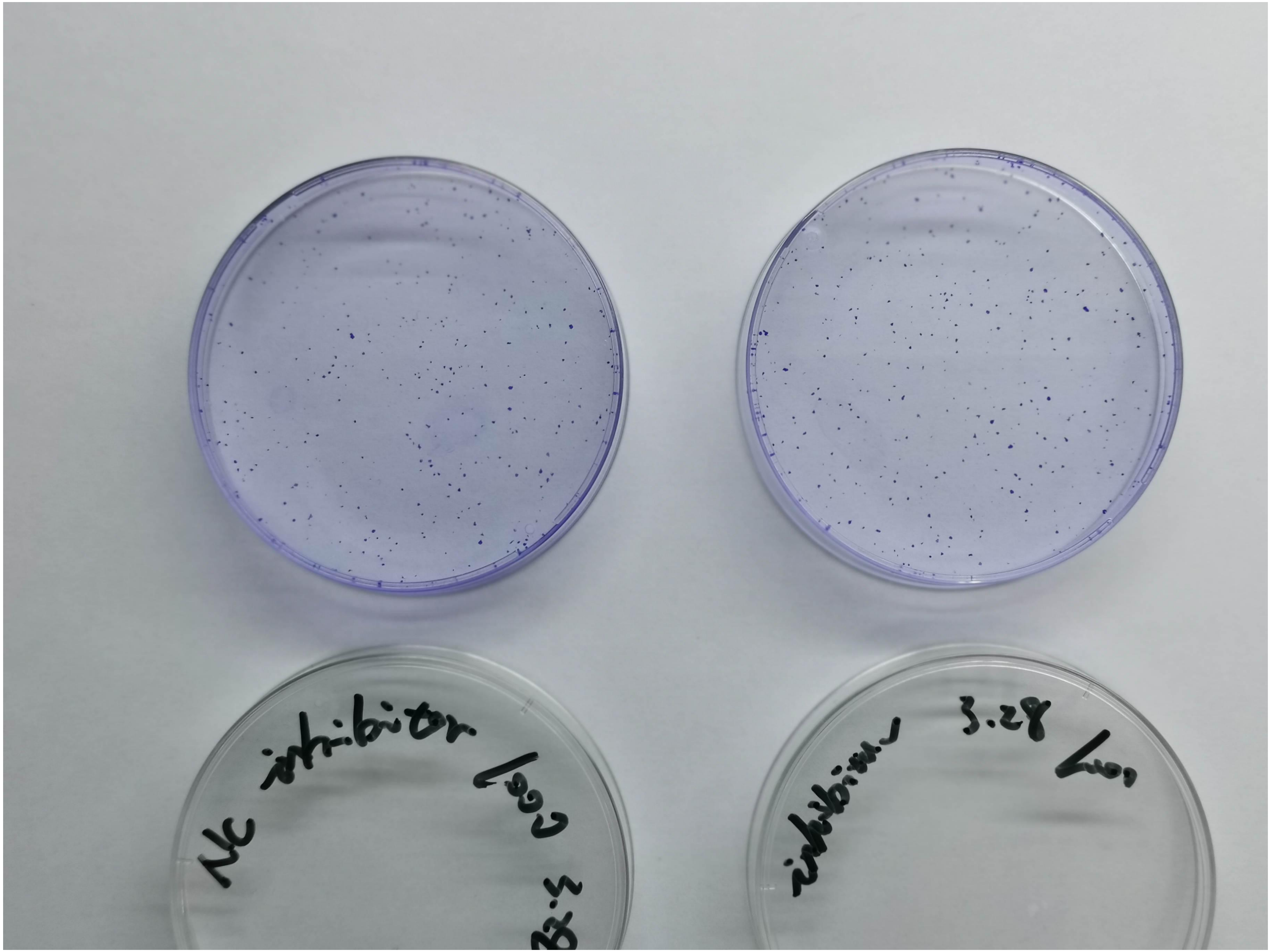
NC inhibitor
3.28

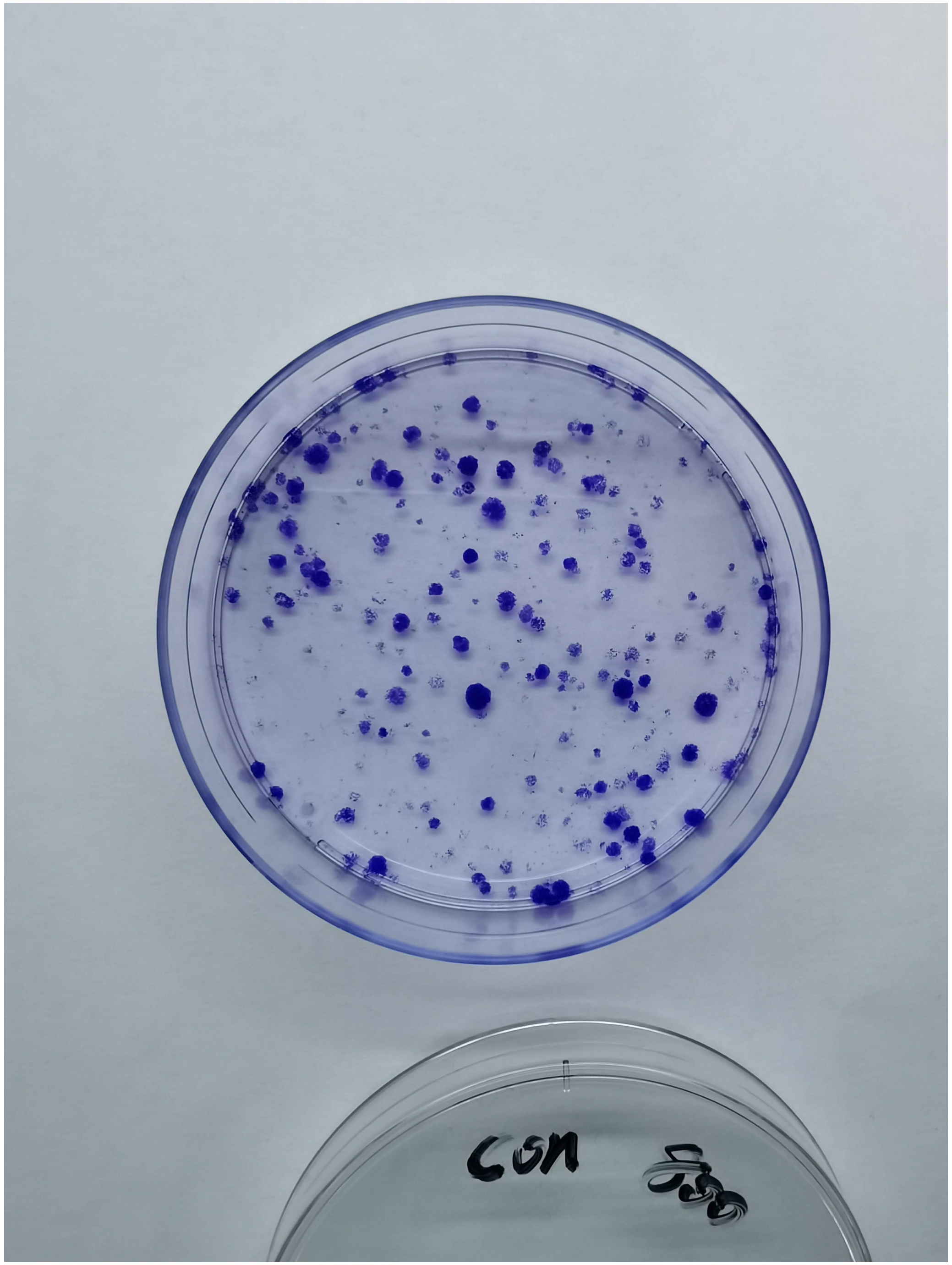
Con

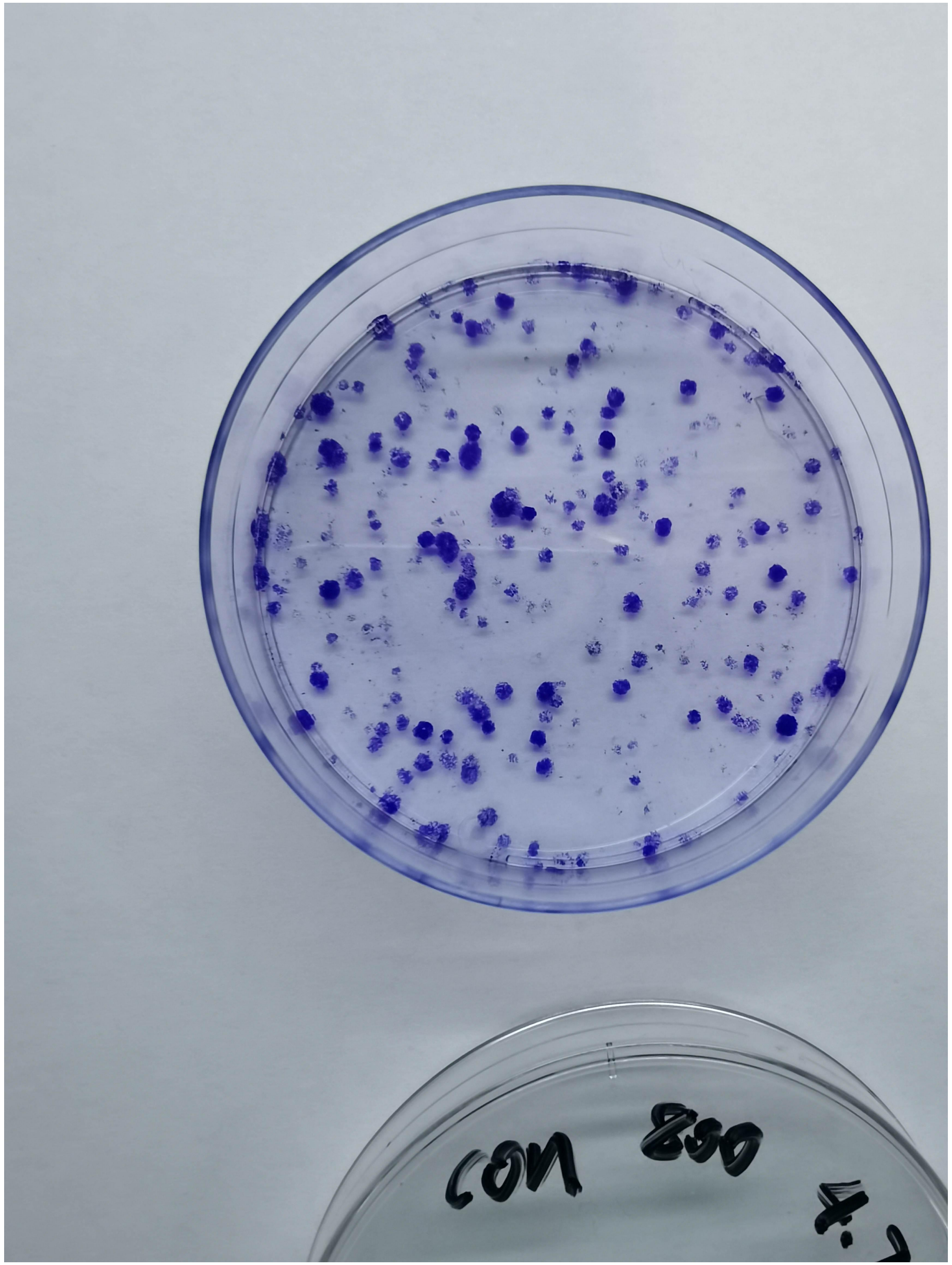
con 850

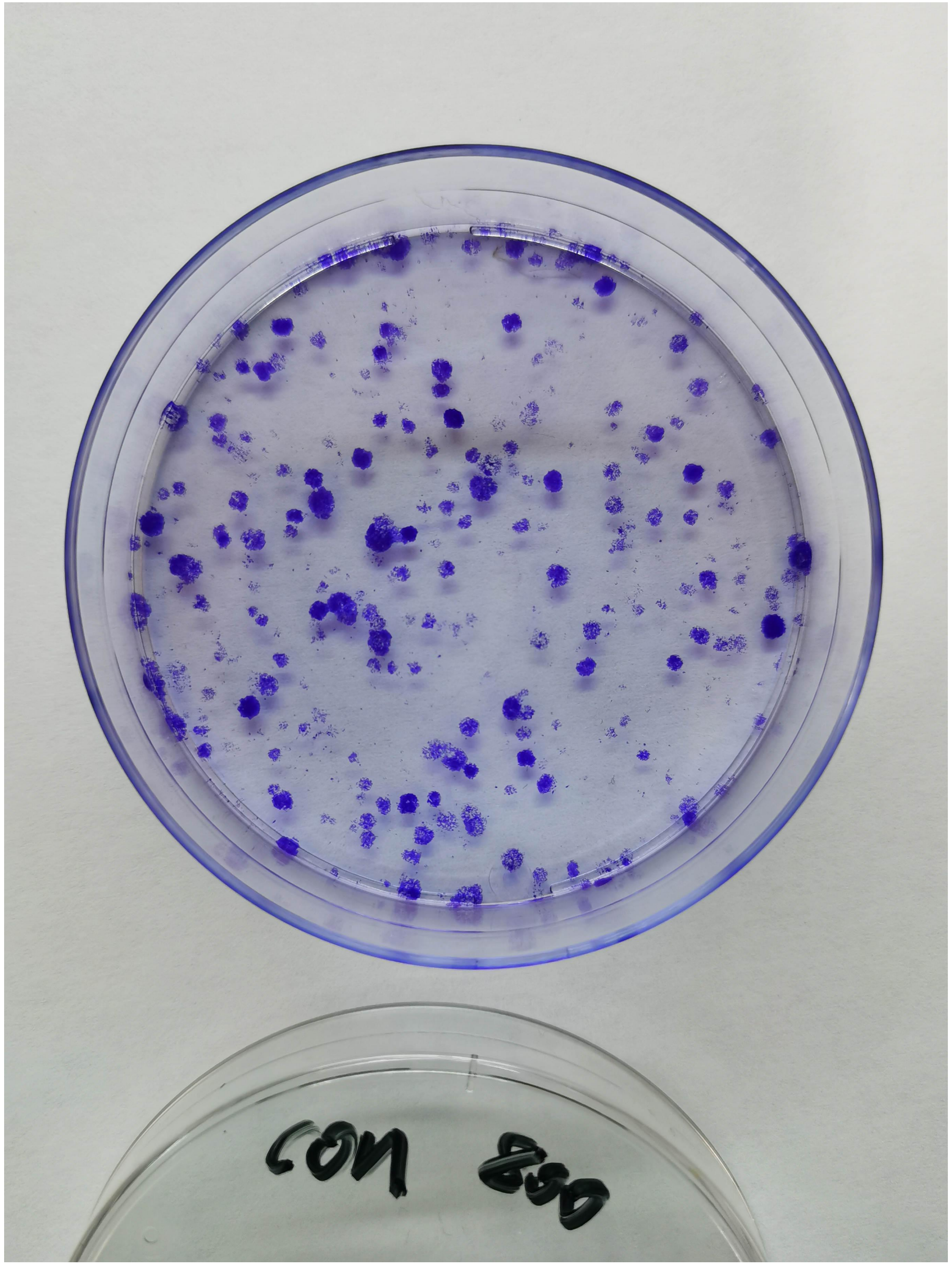
con 850

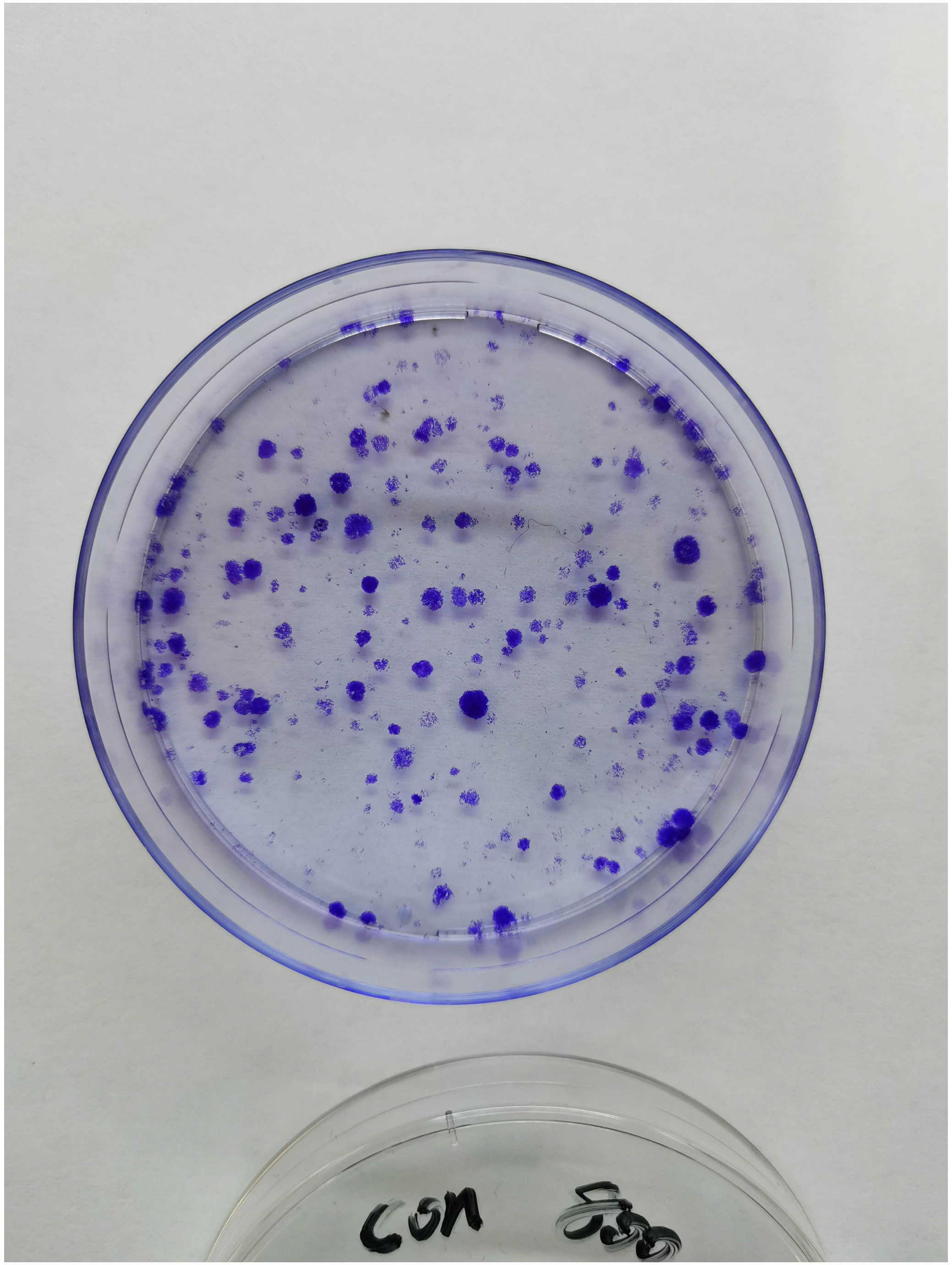
Con

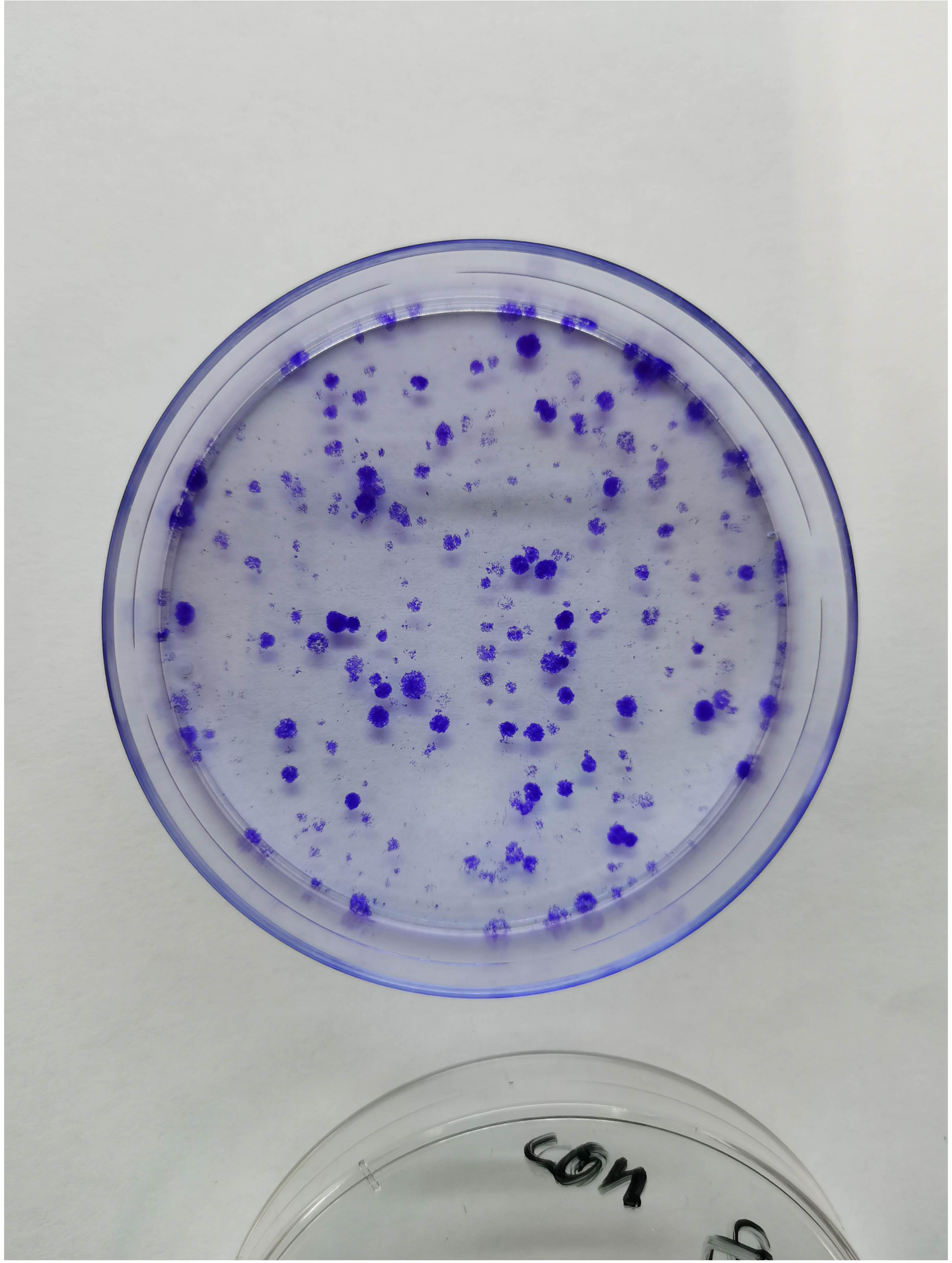

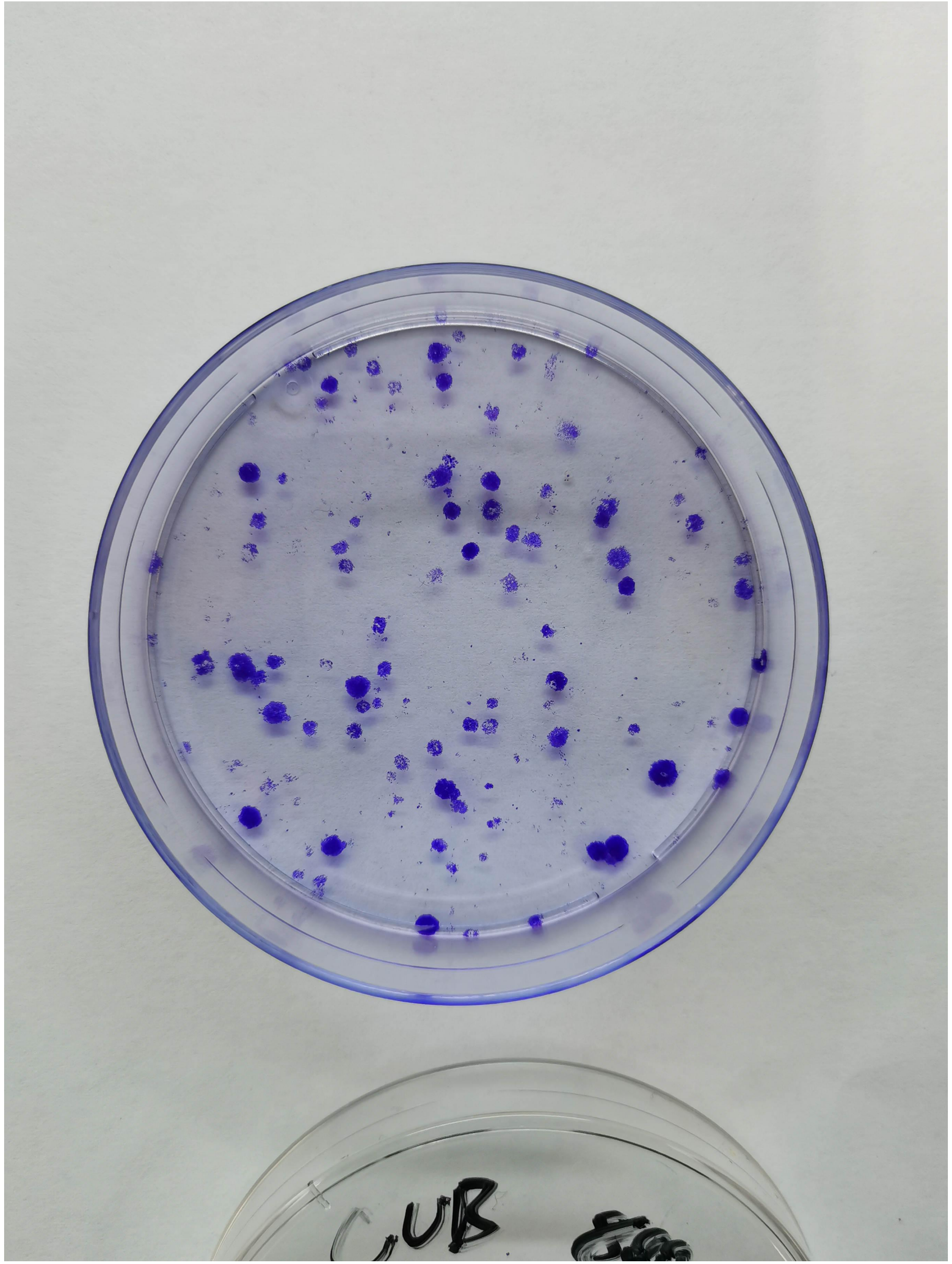

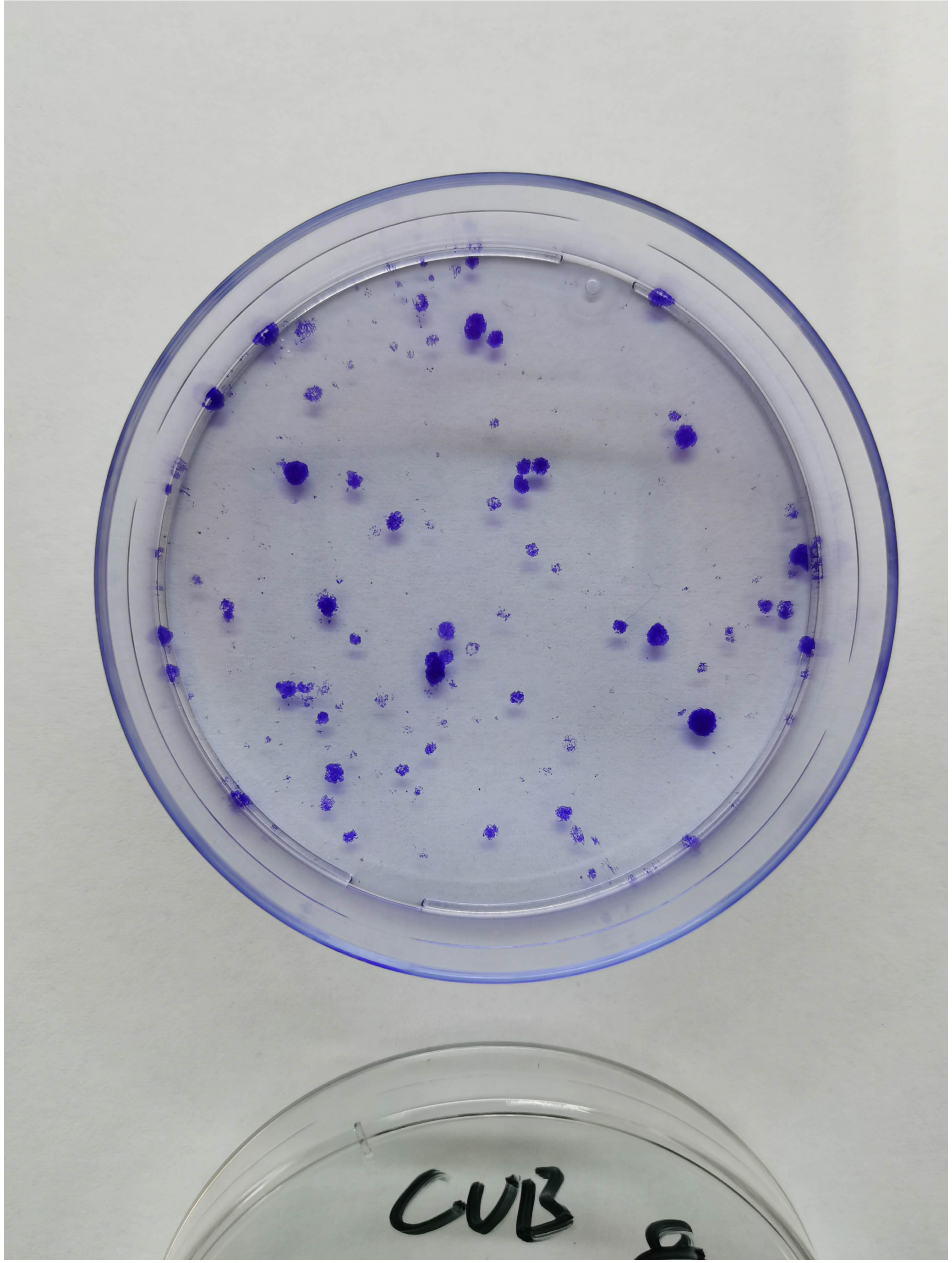
CUB

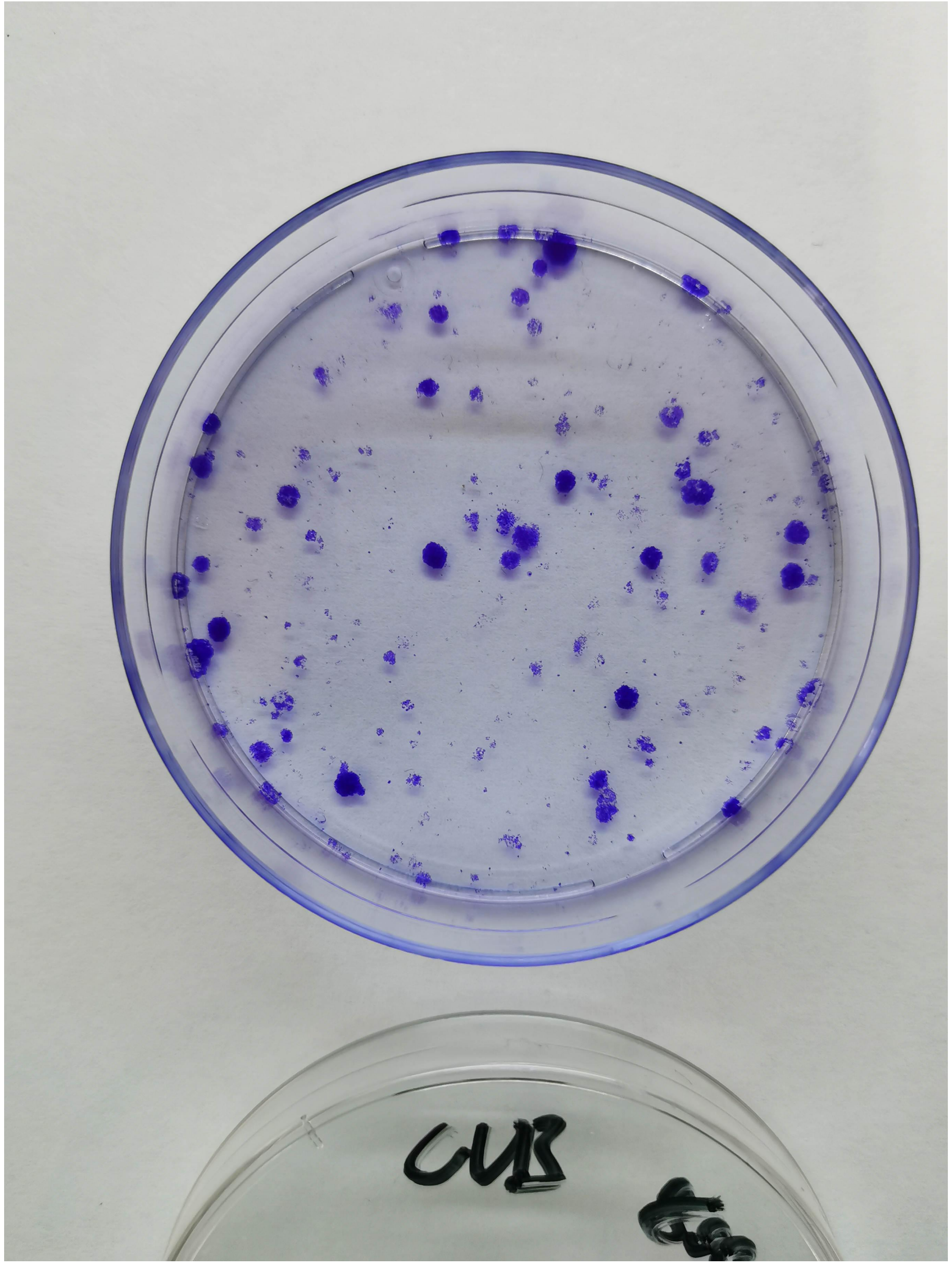

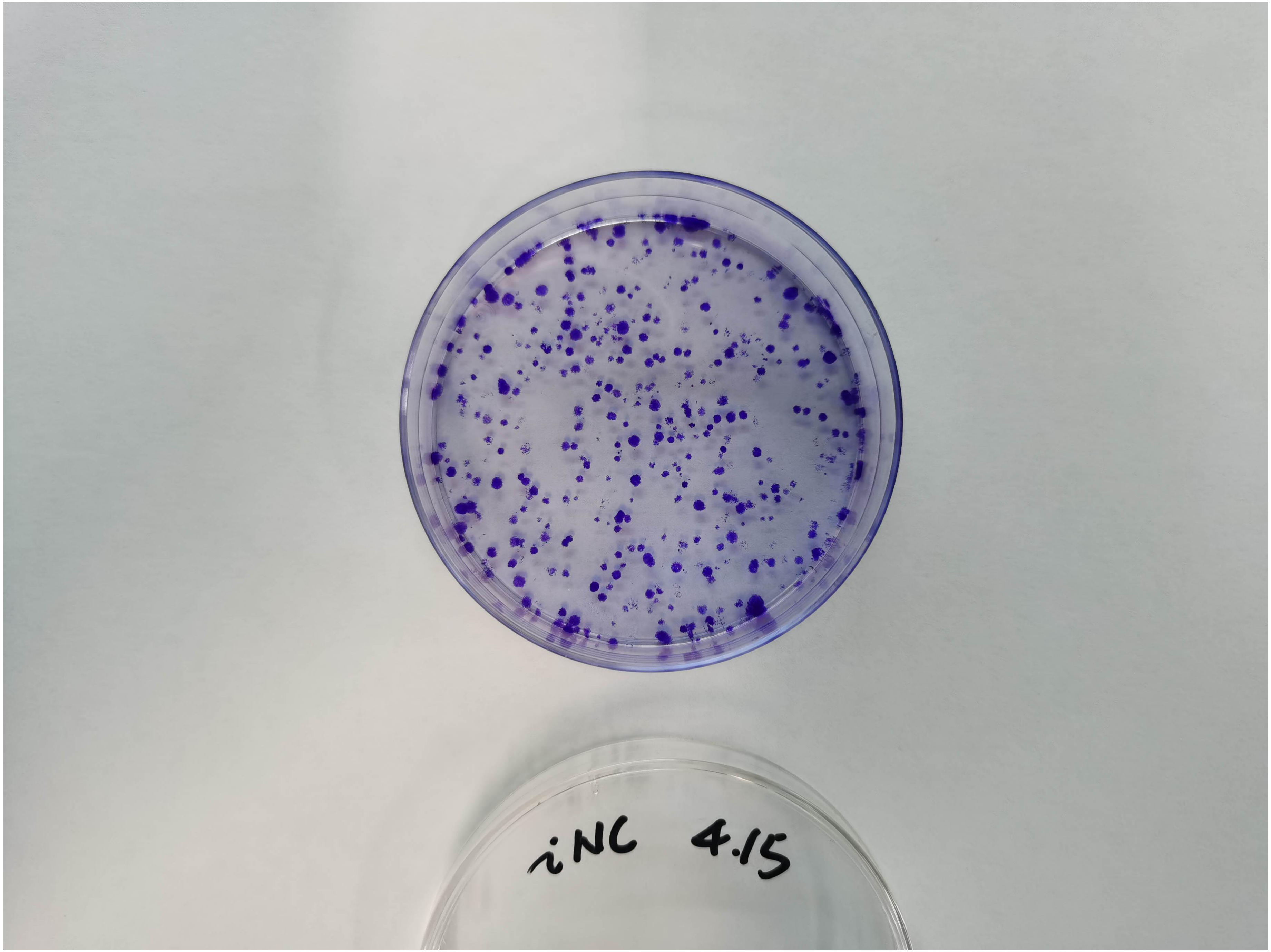
iNC 4.15

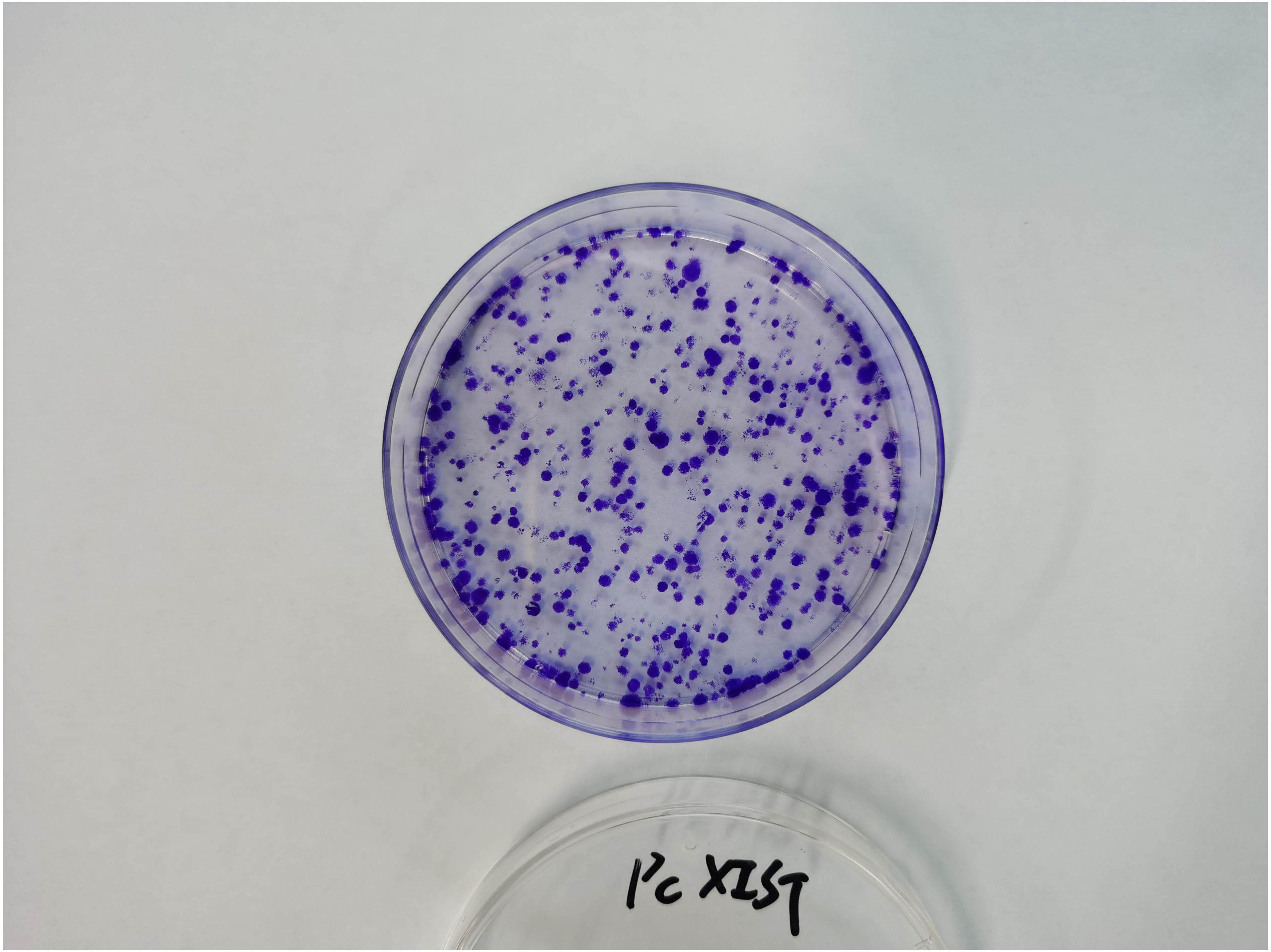
1'c XIST

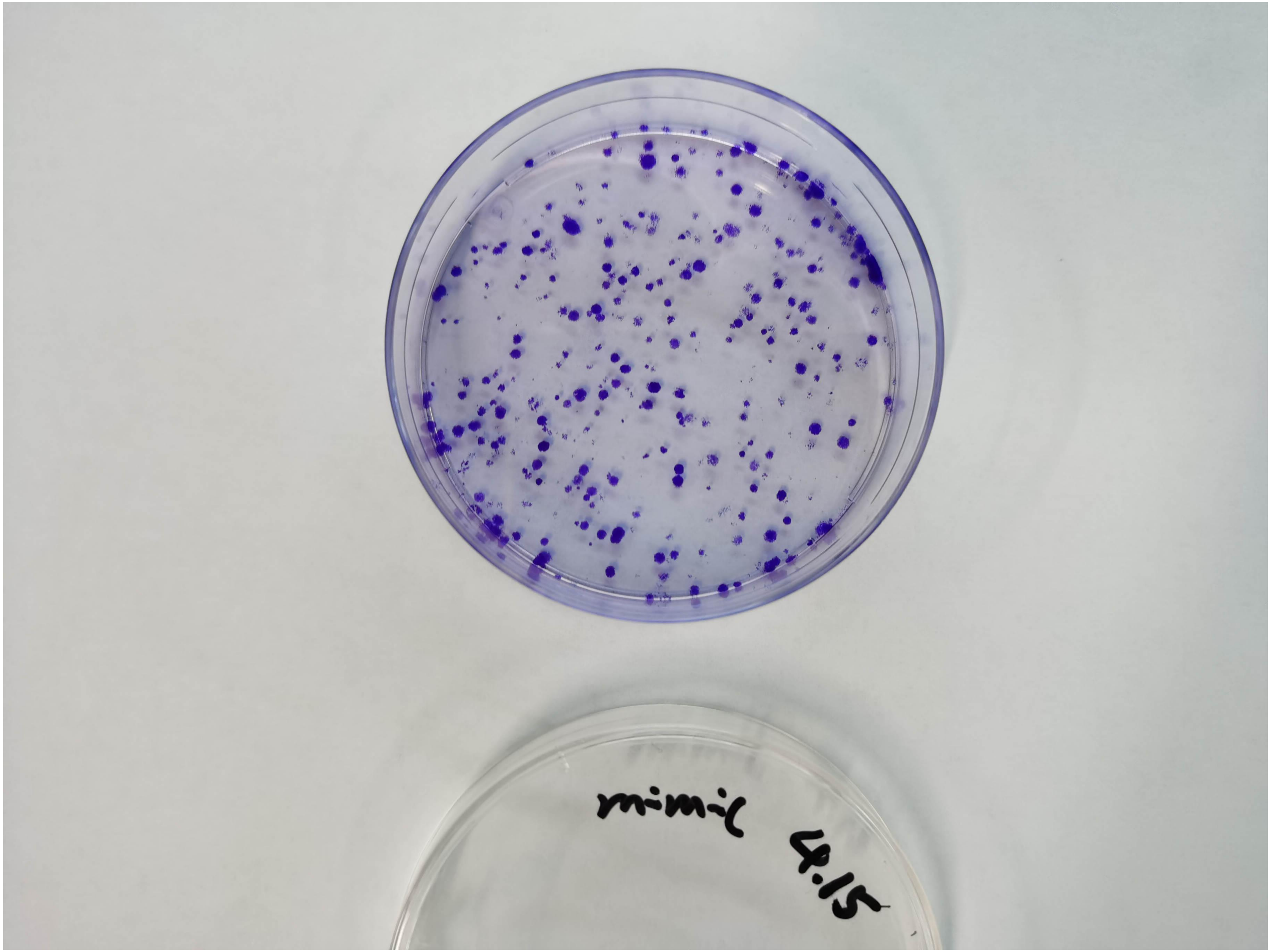
mimic
4.15

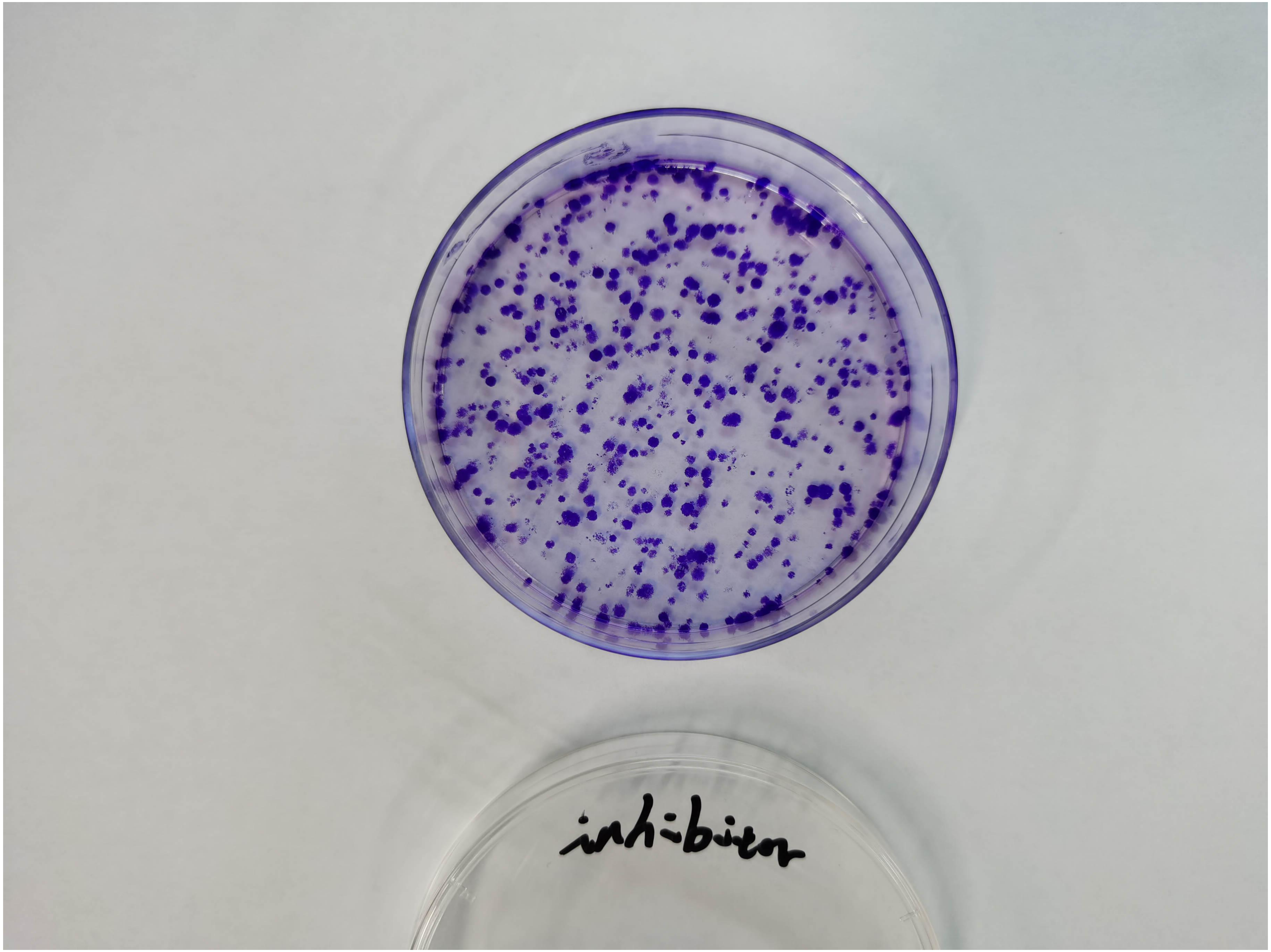
inhibitor

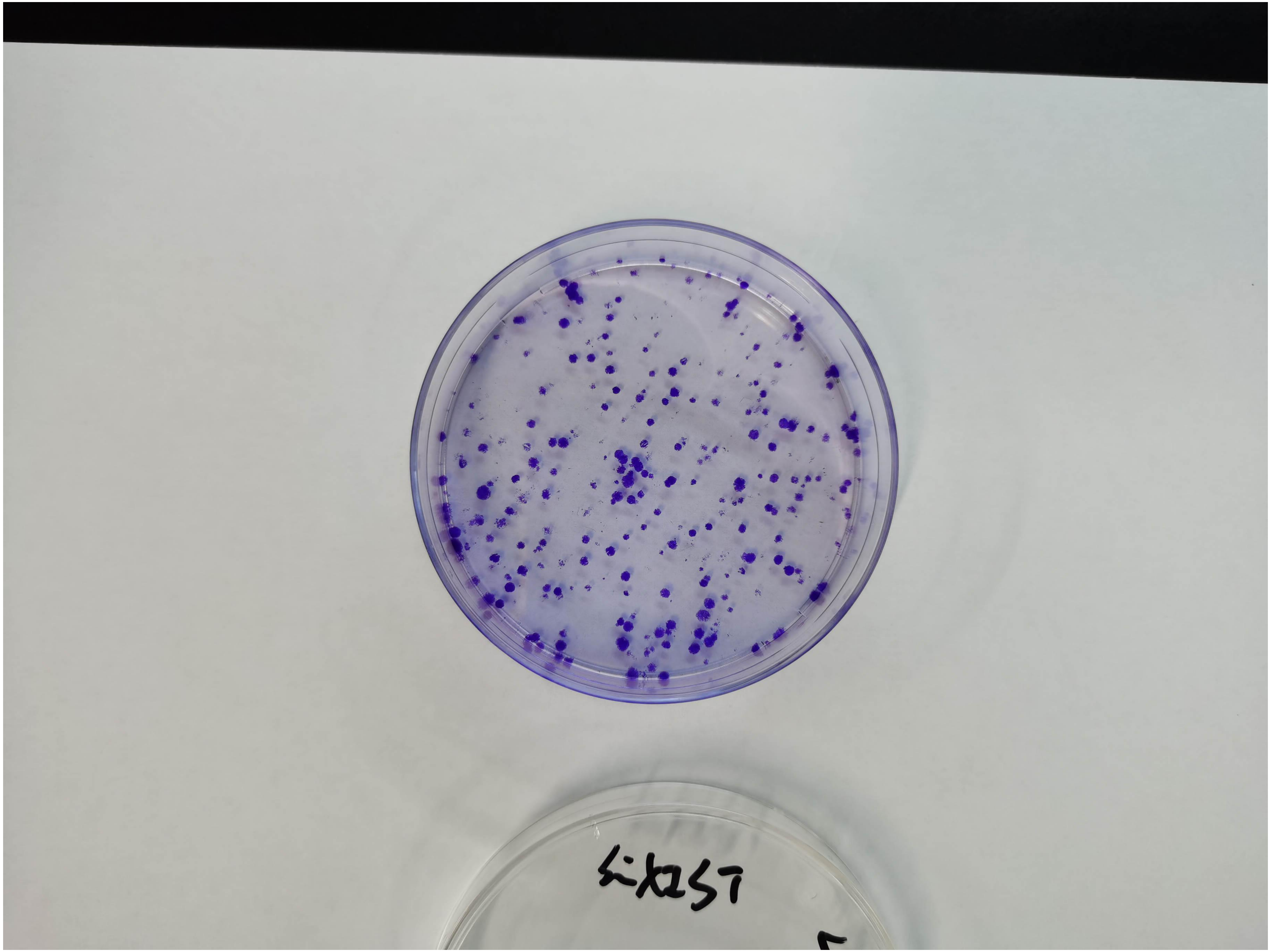

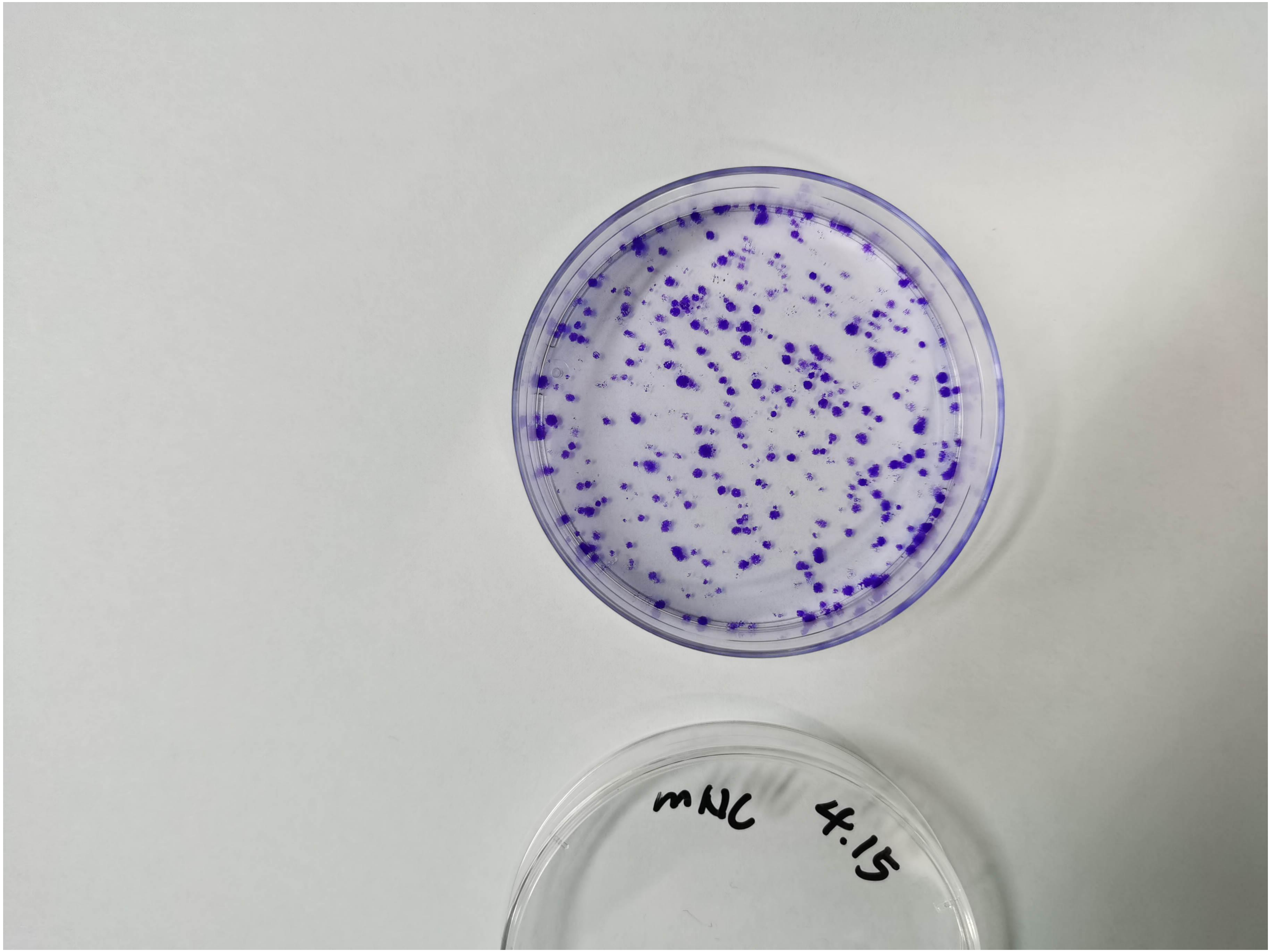
mNC 4.15

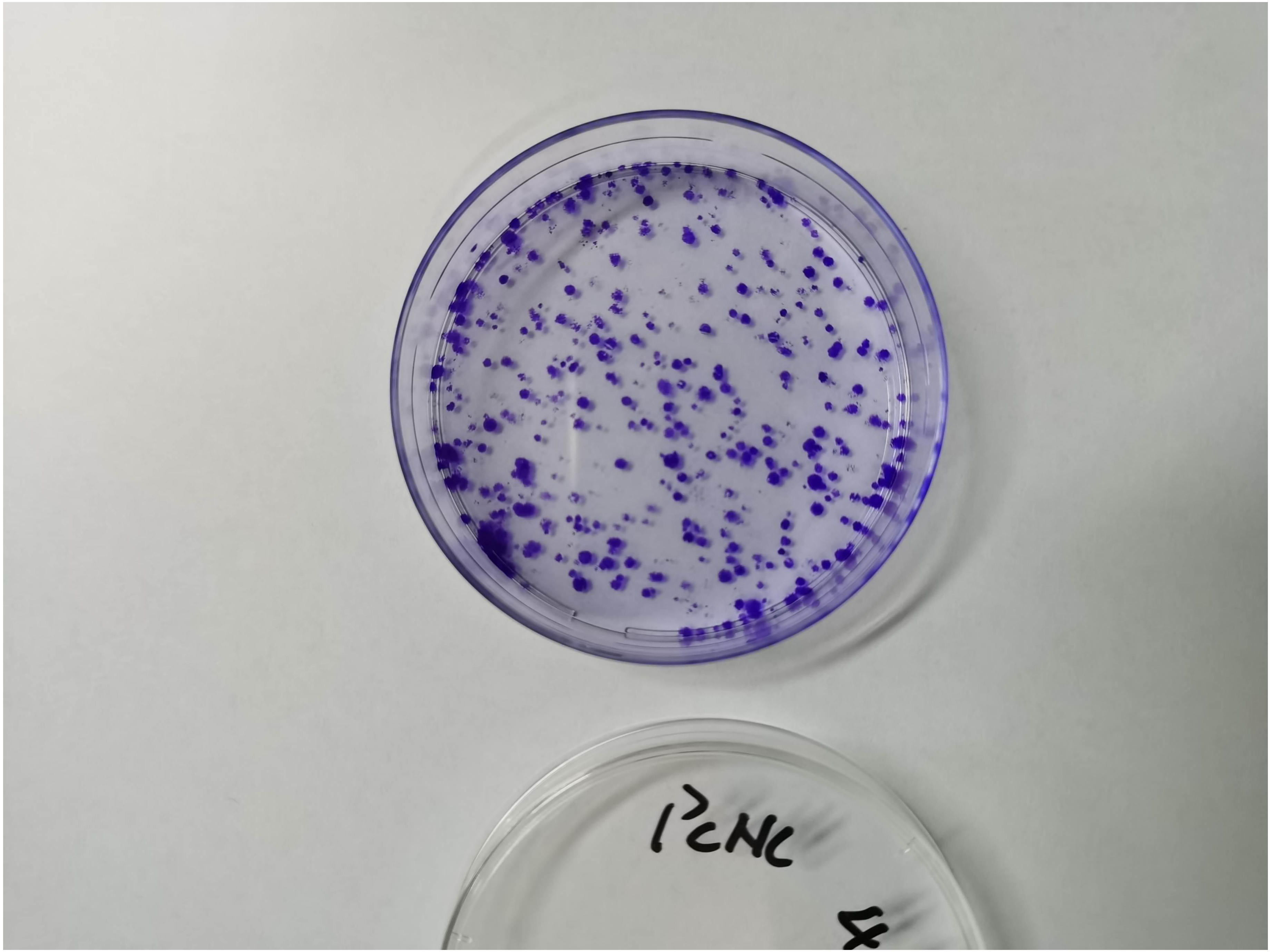

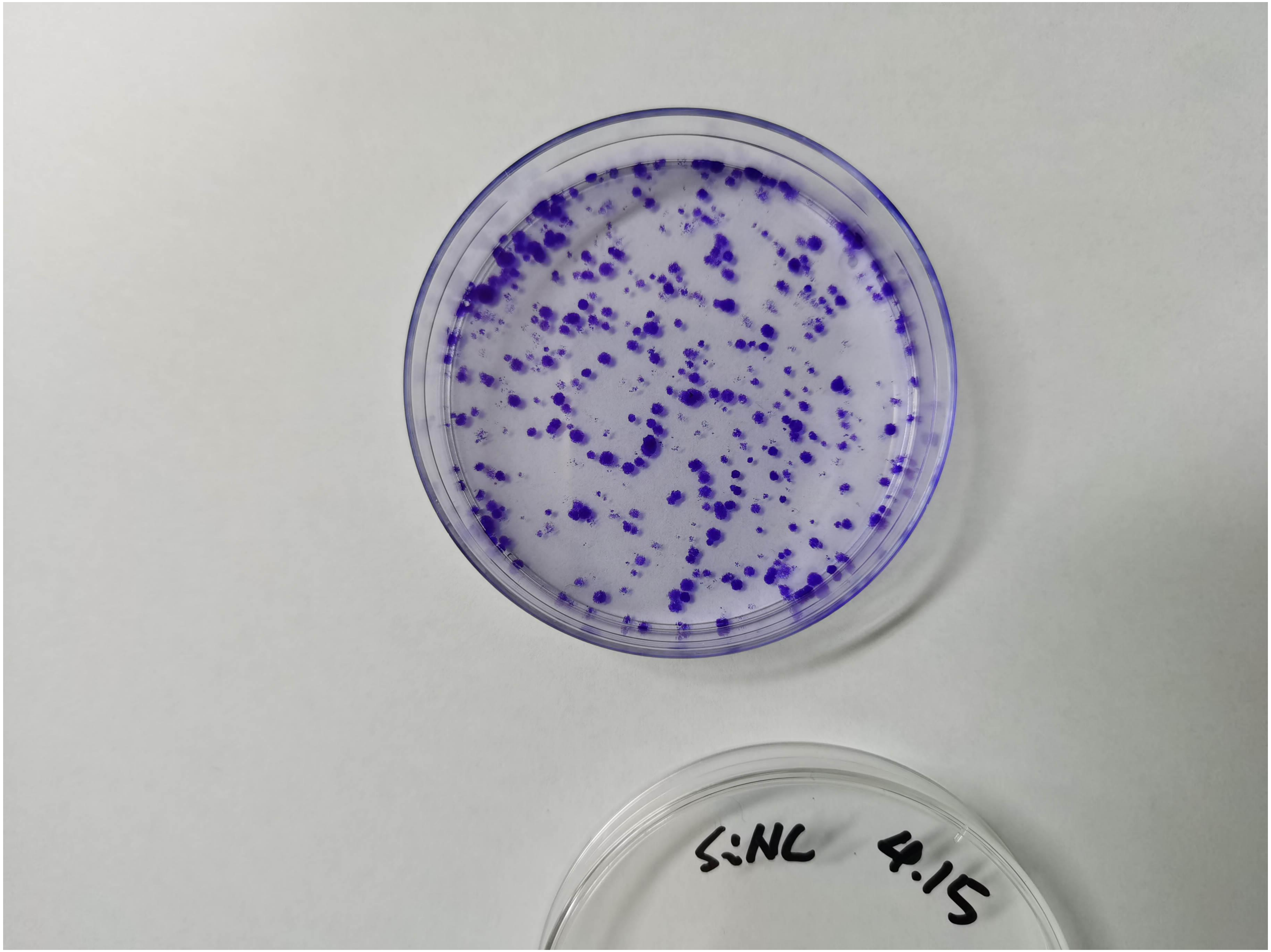
Si:NC 4.15
