# Supplementary material for: Cucurbitacin B Inhibits Cell Proliferation by Regulating X-Inactive Specific Transcript Expression in Tongue Cancer
Source: Front Oncol. 2021 Jul 6;11:651648. doi: 10.3389/fonc.2021.651648 (PMC8290325; doi:10.3389/fonc.2021.651648)
Supplement: Supplementary file 8 [file DataSheet_1.zip › Data Sheet 5.ZIP!/figS6/apoptosis/miR29b-3p-inhibitor-CuB-Layout.pdf]

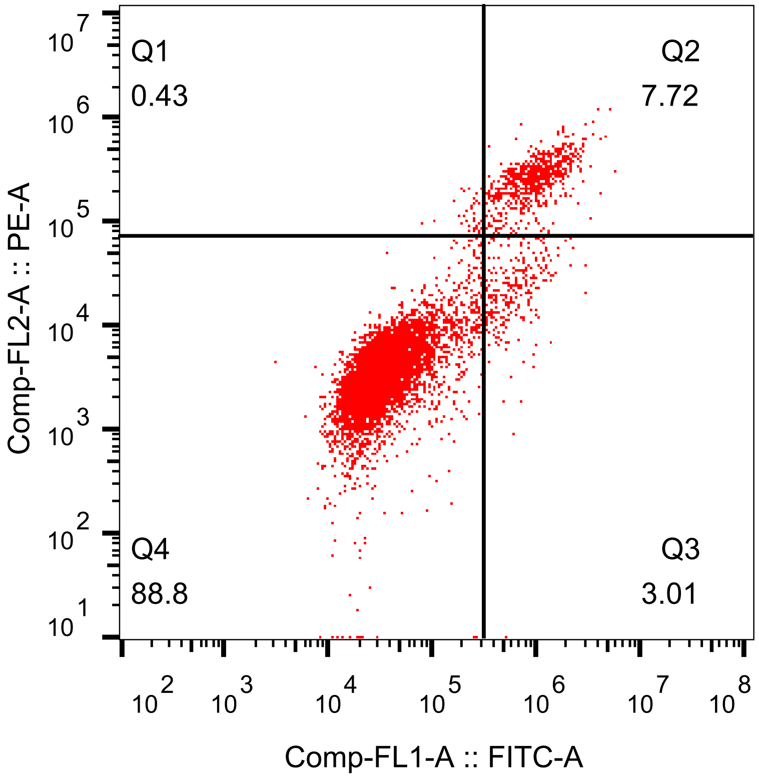

miR29b-3p-inhibitor-CuB-1

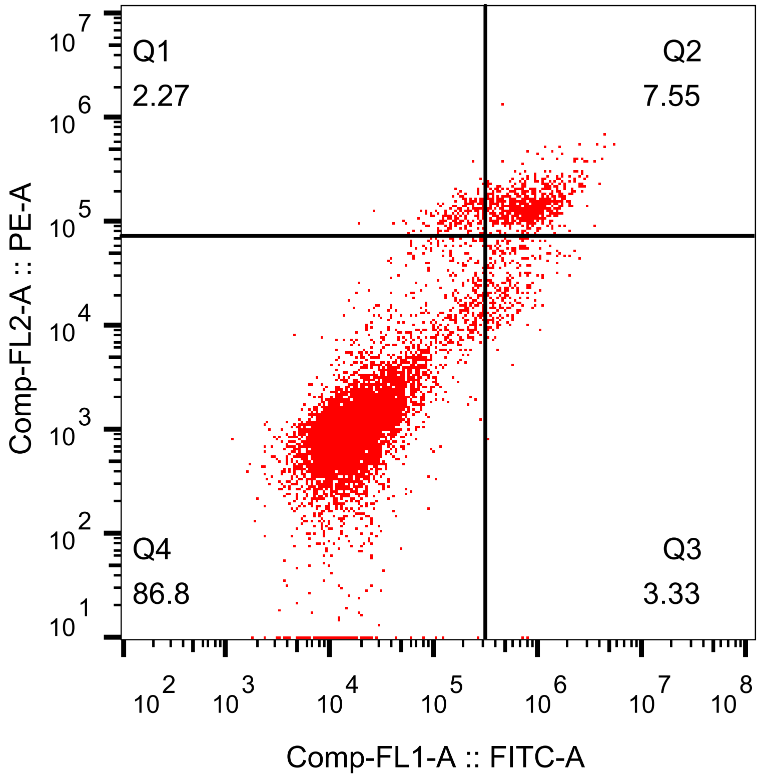

miR29b-3p-inhibitor-CuB-2

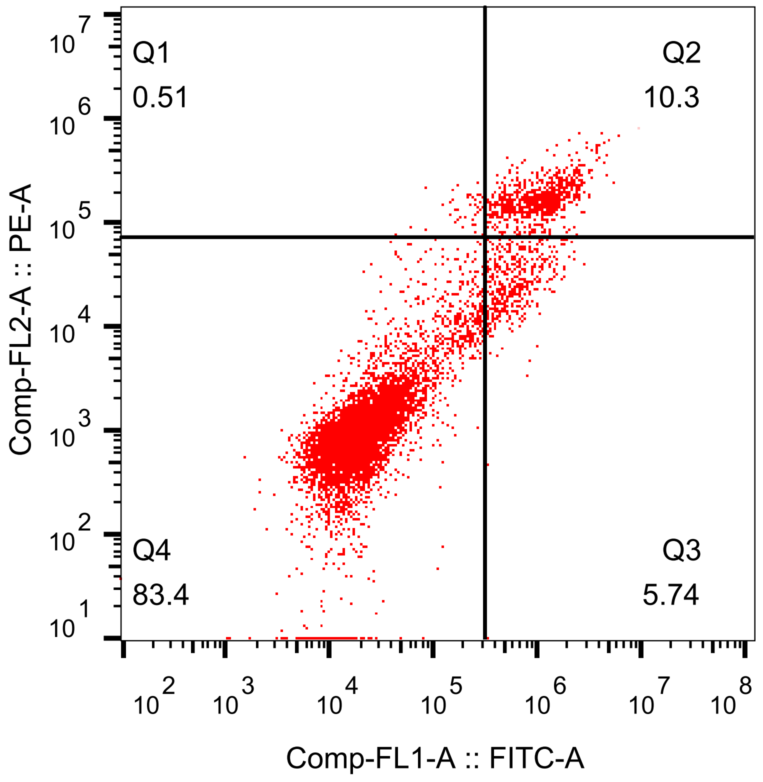

miR29b-3p-inhibitor-CuB-3
